# Melting and density of MgSiO_3_ determined by shock compression of bridgmanite to 1254GPa

**DOI:** 10.1038/s41467-021-21170-y

**Published:** 2021-02-09

**Authors:** Yingwei Fei, Christopher T. Seagle, Joshua P. Townsend, Chad A. McCoy, Asmaa Boujibar, Peter Driscoll, Luke Shulenburger, Michael D. Furnish

**Affiliations:** 1 Earth and Planets Laboratory, Carnegie Institution for Science, Washington, DC USA; 2 Sandia National Laboratories, Albuquerque, NM USA

**Keywords:** Exoplanets, Mineralogy, Mineralogy, Structure of solids and liquids

## Abstract

The essential data for interior and thermal evolution models of the Earth and super-Earths are the density and melting of mantle silicate under extreme conditions. Here, we report an unprecedently high melting temperature of MgSiO_3_ at 500 GPa by direct shockwave loading of pre-synthesized dense MgSiO_3_ (bridgmanite) using the Z Pulsed Power Facility. We also present the first high-precision density data of crystalline MgSiO_3_ to 422 GPa and 7200 K and of silicate melt to 1254 GPa. The experimental density measurements support our density functional theory based molecular dynamics calculations, providing benchmarks for theoretical calculations under extreme conditions. The excellent agreement between experiment and theory provides a reliable reference density profile for super-Earth mantles. Furthermore, the observed upper bound of melting temperature, 9430 K at 500 GPa, provides a critical constraint on the accretion energy required to melt the mantle and the prospect of driving a dynamo in massive rocky planets.

## Abstract (web summary)

The authors here report high melting temperatures of MgSiO_3_ at 500 GPa by direct shockwave loading of pre-synthesized dense bridgemanite. This is essential data to understand the thermal evolution of the interiors of terrestrial (exo-)planets.

## Introduction

The Earth’s lower mantle (extending to a pressure of 136 GPa) is primarily composed of bridgmanite^1^, a dense high-pressure polymorph of MgSiO_3_. MgSiO_3_-bridgmanite further transforms to post-perovskite at the bottom of the lower mantle^2^. Accurate measurements of the density of bridgmanite and post-perovskite as a function of pressure, temperature, and composition, have been the major goal of the Solid-Earth research, using the static and dynamic compression methods^3–10^, in order to decipher the composition of the mantle. However, density measurements of the crystalline phase have been limited to 260 GPa at room temperature and to 170 GPa at high temperature by X-ray diffraction in the diamond-anvil cell^3–6^. The observed melting in the laser-heated diamond-anvil cell up to 62 GPa^11^ and by shockwave methods up to 183 GPa^12,13^ is still debated, because of the extreme temperature required to melt MgSiO_3_-bridgmanite above 60 GPa. Measurements beyond these conditions are unattainable by conventional methods.

The mantle of a rocky exoplanet is likely composed of Mg–Fe silicate based on the observed stellar Fe, Mg, and Si abundances^14,15^. These observations suggest the Mg/Si ratios between 0.67 and 1.5 centered at about 1.0, a MgSiO_3_ composition. Therefore, MgSiO_3_ is an important endmember for all rocky planet mantles. The pressure of the super-Earth mantle could exceed 1400 GPa when its mass reaches 10 Earth masses (*M*_*E*_)^16^. Directly probing materials under such extreme conditions are now attainable only through dynamic compression techniques^17,18^. However, the access to the solid silicate phase along Hugoniot (a shock compression pressure–temperature path) and its melting at higher pressure strongly depends on the initial density of the starting materials.

Typical dynamic compression experiments use MgSiO_3_ enstatite and glass^12,13,19–22^ as the starting materials, with initial densities <3.2 g/cm^3^. These experiments suffer from the complication of phase transitions associated with large density changes (~28%) during shock compression and limited access to the solid phase region because of the fast-rising of Hugoniot temperature with shock pressure. The Hugoniot reaches a melting temperature of MgSiO_3_ below 180 GPa with the low-density starting materials^12,13^. Direct shock compression of pre-synthesized dense MgSiO_3_ has been attempted up to 245 GPa by the traditional two-stage gas-gun technique^9,10^. Very recently, pre-synthesized MgSiO_3_ bridgmanite has been shocked to hot liquid at 1150–1430 GPa by the laser-driven shock technique^18^, but no direct detection of melting was reported, nor was the density of the solid phase along the bridgmanite Hugoniot measured.

In order to obtain density measurements of solid MgSiO_3_ above 245 GPa and detect its melting at much higher pressure, in this study, we synthesized a large polycrystalline bridgmanite sample in a multi-anvil device at 25 GPa and 1673 K, which is the densest recoverable MgSiO_3_ mineral, with a density of 4.1 g/cm^3^ under ambient conditions. The synthesis utilized the newly developed techniques that optimized sample volume at the synthesis condition using extremely large, strong sintered diamond cubes (25.4-mm) (see “Methods”). Using the synthetic MgSiO_3_ bridgmanite as the starting material, we performed magnetically driven plate impact experiments at the Sandia National Laboratories’ Z Pulsed Power Facility. The bridgmanite Hugoniot allows us to measure the density and melting point of the solid phase to a much higher pressure than any previous attempts. These new measurements are essential for developing reliable internal density structure of the solid super-Earth mantles and their thermal evolution.

## Results

### Hugoniot density measurements

Magnetically accelerated flyers launched using the Z Pulsed Power Facility are capable of generating shock pressures over 1000 GPa^23,24^. The plate-impact experiments on the Z machine utilize the impedance-matching technique that allows high-precision determination of the Hugoniot states of the sample through the shock response of a known standard^23^. We carried out a total of 7 plate-impact experiments using the coaxial load geometry, with impact velocities ranging from 9.51 to 30.00 km/s. Pre-synthesized dense MgSiO_3_ bridgmanite samples were used as the starting materials, whose structure of bridgmanite was confirmed by Raman spectra and X-ray diffraction, with a measured unit cell volume of 24.465 ± 0.008 cm^3^/mol (corresponding to a density of 4.103 g/cm^3^). The samples were double-polished parallel to thicknesses from 0.3659 to 0.9339 mm, mounted in a 5-mm Al-ring holder backed by quartz windows, 5-mm square with 1.5-mm thickness.

Supplementary Figure 1 shows the experimental configuration that consists of the north and south sample panels as impacting targets. The impacting target consists of 5-7 square sample holders with an edge length of 5 mm on each north or south panel. A sample diameter of at least 2 mm is required to ensure high-quality data. Each panel contains at least two bridgmanite samples while the remaining sample slots were used for the standards and other samples selected by the Z fundamental science program. Table 1 lists the details of the 7 bridgmanite experiments. Measurements of the four experiments (Z3011, Z3029, Z3116, and Z3203) were made using a symmetric coaxial target with a dual-layer aluminum/copper flyer which resulted in identical impact velocity on the north and south sample panels. Each experiment provides four independent measurements under the same Hugoniot state, producing high-precision data. The experiments (Z3250 and Z3321) at the highest pressures used the same configuration but with an asymmetric A–K gap (Supplementary Fig. 1), which resulted in distinct impacting velocities on the north and south panels. The data precision for Z3250 is still high because three repetition measurements on bridgmanite were made at each panel. One sample on the north panel of the Z3321 shot was compromised, resulting in uncertainty of 2.6% in density measurements, instead of a typical uncertainty of ~1%. The experiment (Z3268) at the lowest pressure (296 GPa) of this study utilized an aluminum (Al) flyer impacting the two bridgmanite samples on the north panel only, producing comparable data quality (Table 1).

**Table 1:** Hugoniot data for MgSiO_3_ using bridgmanite as starting material.

Sample	Flyer	*u*_*f*_ (km/s)	*u*_*p*_ (km/s)	*U*_*s*_ (km/s)	*P* (GPa)	*ρ* (g/cm^3^)	*V*_*P*_ (km/s)	*V*_*B*_ (km/s)
Z3268-N2	Al	12.45 (0.04)	5.11 (0.08)	14.20 (0.15)	297 (5)	6.40 (0.10)		
Z3268-N4	Al	12.35 (0.04)	5.04 (0.07)	14.32 (0.13)	296 (4)	6.32 (0.09)		
**Z3268(average)**			**5.07 (0.05)**	**14.26 (0.10)**	**296 (4)**	**6.36 (0.07)**		
Z3203-N4	Cu	9.51 (0.04)	5.55 (0.05)	15.32 (0.17)	349 (5)	6.43 (0.09)		
Z3203-N6	Cu	9.53 (0.04)	5.59 (0.06)	15.05 (0.27)	345 (5)	6.53 (0.14)		
Z3203-S4	Cu	9.63 (0.04)	5.68 (0.06)	14.87 (0.34)	347 (6)	6.64 (0.18)		
Z3203-S6	Cu	9.64 (0.04)	5.67 (0.06)	15.11 (0.31)	351 (6)	6.56 (0.16)		
**Z3203(average)**			**5.62 (0.03)**	**15.13 (0.13)**	**348 (3)**	**6.52 (0.07)**	**19.38 (1.34)**	**14.12 (1.90)**
Z3116-N4	Cu	10.90 (0.04)	6.43 (0.07)	16.01 (0.41)	423 (8)	6.85 (0.21)		
Z3116-N6	Cu	10.91 (0.04)	6.46 (0.06)	15.83 (0.38)	419 (8)	6.93 (0.20)		
Z3116-S4	Cu	10.90 (0.04)	6.47 (0.06)	15.73 (0.37)	417 (7)	6.97 (0.20)		
Z3116-S6	Cu	10.93 (0.04)	6.43 (0.06)	16.17 (0.30)	427 (7)	6.81 (0.15)		
**Z3116(average)**			**6.45 (0.03)**	**15.95 (0.18)**	**422 (4)**	**6.88 (0.09)**	**20.15 (2.35)**	**14.86 (0.85)**
Z3029-N4	Cu	12.20 (0.04)	7.24 (0.07)	16.86 (0.19)	500 (6)	7.19 (0.11)	18.28 (1.94)	
Z3029-N6	Cu	12.25 (0.04)	7.31 (0.08)	16.58 (0.51)	497 (11)	7.34 (0.28)	17.95 (2.02)	
Z3029-S4	Cu	12.21 (0.04)	7.25 (0.09)	16.82 (0.77)	500 (15)	7.23 (0.40)	17.68 (1.25)	
Z3029-S6	Cu	12.29 (0.04)	7.30 (0.10)	16.86 (1.00)	505 (19)	7.26 (0.51)	16.88 (1.56)	
**Z3029(average)**			**7.27 (0.04)**	**16.80 (0.21)**	**500 (5)**	**7.24 (0.12)**	**17.65 (0.82)**	**15.39 (1.89)**
Z3011-N4	Cu	15.24 (0.04)	9.01 (0.08)	19.65 (0.31)	726 (10)	7.58 (0.16)		
Z3011-N6	Cu	15.24 (0.04)	9.10 (0.08)	19.03 (0.18)	710 (8)	7.86 (0.12)		
Z3011-S4	Cu	15.24 (0.04)	9.08 (0.09)	19.19 (0.50)	714 (13)	7.79 (0.26)		
Z3011-S6	Cu	15.23 (0.04)	9.08 (0.08)	19.14 (0.26)	712 (10)	7.80 (0.15)		
**Z3011(average)**			**9.07 (0.04)**	**19.22 (0.14)**	**715 (5)**	**7.76 (0.08)**		**17.35 (1.10)**
Z3250-S2	Cu	16.96 (0.04)	10.11 (0.09)	20.50 (0.13)	850 (9)	8.09 (0.11)		
Z3250-S4	Cu	17.02 (0.04)	10.15 (0.09)	20.54 (0.15)	855 (10)	8.11 (0.11)		
Z3250-S6	Cu	17.04 (0.04)	10.13 (0.09)	20.79 (0.23)	864 (11)	8.00 (0.14)		
**Z3250S(average)**			**10.13 (0.05)**	**20.58 (0.09)**	**856 (6)**	**8.07 (0.07)**		**18.07 (0.78)**
Z3250-N2	Cu	17.68 (0.04)	10.53 (0.09)	21.18 (0.12)	914 (10)	8.16 (0.10)		
Z3250-N4	Cu	17.64 (0.04)	10.53 (0.09)	20.98 (0.17)	906 (10)	8.23 (0.12)		
Z3250-N6	Cu	17.62 (0.04)	10.51 (0.09)	21.06 (0.21)	907 (11)	8.18 (0.14)		
**Z3250N(average)**			**10.52 (0.05)**	**21.09 (0.09)**	**909 (6)**	**8.19 (0.07)**		**20.01 (1.24)**
Z3321-S2	Al	29.29 (0.04)	12.52 (0.17)	23.43 (0.45)	1202 (22)	8.81 (0.27)		
Z3321-S4	Al	29.26 (0.04)	12.41 (0.17)	23.82 (0.40)	1212 (21)	8.56 (0.24)		
**Z3321S(average)**			**12.47 (0.12)**	**23.64 (0.30)**	**1207 (15)**	**8.68 (0.18)**		
Z3321-N2	Al	North 2 sample compromised						
Z3321-N4	Al	30.00 (0.04)	**12.83 (0.19)**	**23.86 (0.15)**	**1254 (29)**	**8.87 (0.23)**		

Bold values indicate average values.

Velocity histories were measured by a velocity interferometer system for any reflector (VISAR)^25^. We used 38 channels of VISAR on each plate-impacting experiment, with fringe sensitivities ranging from 277 to 2293 m/s/fringe. Typically, data were recorded in three channels for each bridgmanite sample. The VISAR probe accurately tracks the velocity of the flyer from rest to impact and records the shocking history in the bridgmanite sample and the quartz window. The bridgmanite shock velocity (*U*_*s*_) was determined from the transit time for the shock to traverse the sample. The uncertainties in shock velocity are less than 2% based on the uncertainty in the transit time <0.7 ns. The particle velocity (*u*_*p*_) and associated uncertainty were determined through impedance matching with the copper and aluminum flyer plate in a Monte Carlo technique which varied the equation of state of the flyer by ±3% and the flyer velocity, bridgmanite shock velocity, and bridgmanite initial density within 1%. Table 1 lists the measured shock and particle velocities, also shown in Supplementary Fig. 2. A least-squares fit to the data yielded a linear *U*_*s*_–*u*_*p*_ relation, *U*_*s*_ = 7.922(±0.139) + 1.249(±0.015)*u*_*p*_.

**Fig. 1:**
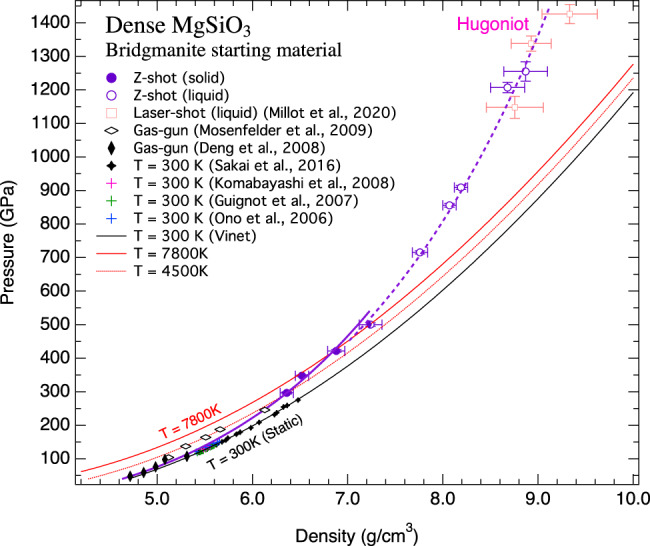
Hugoniot data obtained using dense MgSiO_3_-bridgmanite as the starting material. The pressure–density data from magnetically driven shock wave experiments in this study are shown with the purple circles. The orange open squares represent liquid MgSiO_3_ Hugoniot data by the laser-driven shock^18^. The black solid and open diamonds represent compression data by the gas-gun method^9,10^. The static compression data for MgSiO_3_ post-perovskite at 300 K^3–6^ are also shown for comparison. The purple solid and dashed curves are the calculated Hugoniots for post-perovskite and liquid, respectively, by DFTMD simulations. The calculated isotherms at 300, 4500, and 7800 K are shown with solid black, dashed red, and solid red curves, respectively.

Figure 1 shows the density of MgSiO_3_ as a function of pressure up to 1254 GPa along the Hugoniot. The Hugoniot data of the bridgmanite with the initial density of 4.10 g/cm^3^ are listed in Table 1. Over the experimental pressure range, the density increases more than twice from the initial bridgmanite. Under static compression, MgSiO_3_-bridgmanite transforms to post-perovskite at ~125 GPa, with only about 1% density change^2^. Our experiments are expected to have shocked the sample into the stability field of post-perovskite and then into a liquid state along the Hugoniot. We assumed the measured densities of the solid phase reflect those of post-perovskite because our experiment at the lowest pressure (296 GPa) has reached the conditions significantly above the bridgmanite-ppv transition boundary along with hot Hugoniot temperature. In order to complement experimental data, we performed a series of ab initio density functional theory (DFT) calculations for the bridgmanite, post-perovskite, and liquid phases of MgSiO_3_ (see “Methods”). Results are summarized in Supplementary Table 1. Our DFT-based molecular dynamics (DFTMD) calculations follow a similar approach as in the previous work^17^. The calculated density–pressure relation for post-perovskite along Hugoniot is shown by the solid purple curve which is in excellent agreement with the three low-pressure shots at 296, 348, and 422 GPa (Fig. 1), where the samples remained solid (see sound velocity data below). The calculated liquid Hugoniot pressure–density relation is shown by the dashed purple curve which is again in excellent agreement with the Hugoniot data between 500 and 1225 GPa. There is no liquid–liquid transition that can be detected from the liquid density data. The recent liquid Hugoniot data at 1150–1430 GPa by the laser-driven shock technique^18^ are also shown for comparison. Within the uncertainties of the data, the laser-shock data are also plotted on the calculated curve. At the low-pressure end (<245 GPa), the gas-gun data^9,10^, which were produced at the limit of the sample size, are also within the general trend.

### Sound velocity and melting temperature determination

**Fig. 2:**
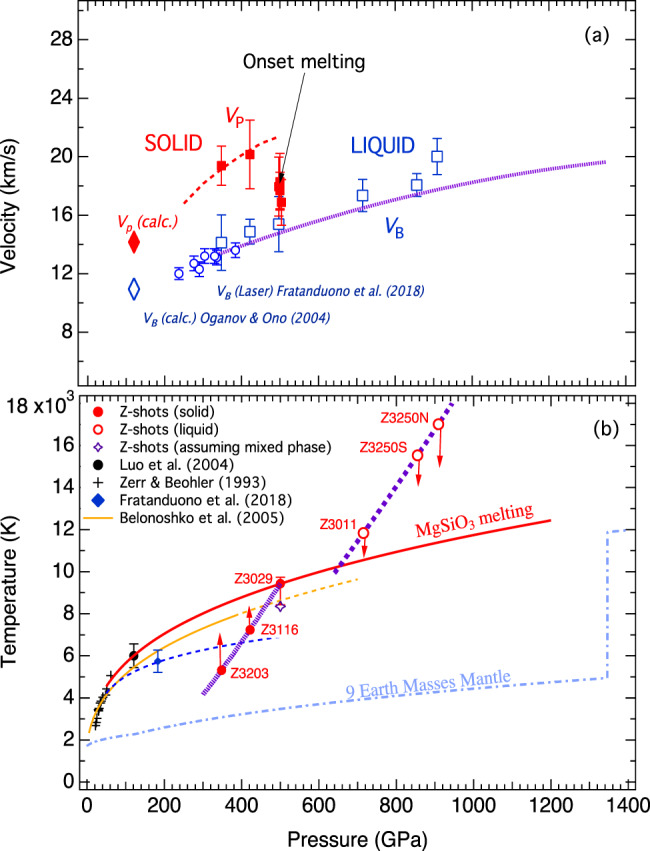
Comparision of the measured sound velocities and melting temperature of MgSiO_3_ at high pressure with results from previous studies. **a** The bulk sound velocities (*V*_*B*_) along Hugoniot are shown by the blue open squares, compared with theoretical calculations of this study (purple dotted line). The measured bulk sound velocities of liquid MgSiO_3_ enstatite target by laser-driven shock^13^ are also plotted for comparison. The longitudinal velocities (*V*_*p*_) of the solid phase are shown by the red solid squares. The calculated values of *V*_*p*_ and *V*_*B*_ for post-perovskite at 125 GPa^27^ are also shown for comparison. **b** The Z shots supporting longitudinal wave (red solid circles) are plotted on the calculated Hugoniot for solid post-perovskite (thick purple dotted line) whereas the experiments shocked into a liquid are plotted on the calculated liquid Hugoniot (thick purple dashed line). The red curve represents the melting curve fitted to the upper bound melting constraint at 500 GPa along with low-pressure constraints from gas-gun compression of glass (black solid circle)^12^ and static experiment (crosses)^11^. The vertical red arrows point toward melting temperatures. The open purple star indicates melting temperature assuming the shock entering a mixed phase. Theoretically calculated melting curve (yellow line)^28^ and estimated melting point (blue diamond) and curve (blue dashed line) from laser-shock of enstatite^13^ are shown for comparison. The temperature profile of super-Earth with 9 *M*_E_ used for density profile calculation is also shown.

The DFTMD calculations define the Hugoniot temperature and pressure relations for the post-perovskite and the liquid phases (Supplementary Table 1). By determining the physical states (solid or liquid) of the shocked bridgmanite, we constrain the melting temperature of MgSiO_3_ at extreme pressure. The standard way to detect melting in a dynamic compression experiment is via the sound velocities and the analysis of the release wave profiles. We determined the sound velocities using the overtaking rarefaction method with quartz windows^24,26^. In the overtaking rarefaction method, the back-propagating wave in the Cu impactor reflects from the Cu/Al interface as a release wave, which then propagates forward through the Cu impactor, bridgmanite sample, and quartz window until it overtakes the initial shock wave (Supplementary Fig. 3) (see “Methods”). The use of quartz windows decreases the uncertainty in the overtaking time and enables additional analysis of the wave profile. Table 1 summarizes the results from the sound velocity analyses. Supplementary Fig. 4 shows representative wave profiles, including a single-stage bulk release (plastic unloading) in liquid, a profile containing both longitudinal and bulk releases in the solid phase, and a complex profile showing edge wave in addition to the longitudinal and bulk releases in solid. The experiments (Z3011 at 715 GPa, Z3250S at 856 GPa, and Z3250N at 909 GPa) show simple single-stage bulk release. We analyzed the sound velocity of these shots using a multi-sample technique which leverages that the different bridgmanite sample thicknesses produced different window overtake times. The measured sound velocities are consistent with the calculated bulk sound velocity of liquid (Fig. 2a). The calculated Hugoniot temperatures of these experiments are above 11,800 K (Supplementary Table 1) and they are expected to be shocked into the liquid state (Fig. 2b).

The two experiments (Z3203 at 348 GPa and Z3116 at 422 GPa) show both longitudinal and bulk release waves. The measured bulk sound velocities agree well with the calculated bulk sound velocities along Hugoniot (Fig. 2a). We determined the longitudinal sound velocities of these two experiments from the longitudinal release profile using the single-sample technique^24^. The values of the longitudinal sound velocity are about 36% higher than the bulk sound velocities because of the shear component of the solid. Our data extrapolated to low pressure compare favorably to previous first-principles calculations^27^.

The results from experiment Z3029 at 500 GPa are particularly interesting. All wave profiles show longitudinal release (Supplementary Fig. 5). Analysis of one bulk release indicates that the bulk sound velocity of this shot is consistent with the predicted value. However, the derived longitudinal velocities are substantially lower than the expected value for solid but larger than the bulk sound velocity (Fig. 2a). The decrease in sound velocity is caused by shear softening likely related to melting, indicating an onset of melting.

For several wave profiles, the initial release can be attributed to an edge rarefaction impinging on the shock front in the center of the target prior to overtaking of the longitudinal wave (Supplementary Figs. 4c and 5). We find no viable explanation other than an edge wave for the first stage of a three-stage release because if it were a longitudinal wave, the velocity would be unrealistically large and the flyer characteristics would not result in an initial wave prior to the release from the Cu/Al interface. The edge wave effect typically causes a systematic overestimate of the sound velocity (see “Methods”) by about 3.5%. This effect could explain the systematic offset between the observed and calculated sound velocities (Fig. 2a). Our calculated bulk sound velocities are also in good agreement with the measured bulk sound velocities of liquid MgSiO_3_ at lower pressures by laser-shock technique, using enstatite as the target^13^.

The temperatures of the shockwave experiments were not experimentally measured because the samples were not sufficiently transparent. We rely on DFTMD calculations (see “Methods”) to estimate Hugoniot temperatures at the shock pressures. The calculated Hugoniot temperatures at 500 GPa (shot Z3029) are 9430 K for post-perovskite and 6520 K for the liquid phase. The shocked bridgmanite is definitively at solid-state at 422 GPa (shot Z3116), corresponding to a Hugoniot temperature of 7240 K, which is higher than the extrapolated melting temperature from laser-shock of enstatite^13^, but lower than the calculated melting curve^28^. The liquid state is achieved at 715 GPa (shot Z3011), corresponding to a Hugoniot temperature of 11,800 K. The wave profiles of the 500-GPa shot (Supplementary Fig. 5) show a clear longitudinal wave, indicating a solid phase component. By placing the shot along the solid Hugoniot, we obtain a melting temperature of MgSiO_3_ at about 9430 ± 600 K (Fig. 2). The inferred melting temperature would represent an upper bound. From the deduced sound velocity data, there is a decrease in longitudinal sound velocity (Fig. 2a). In the absence of structural measurement, we do not have the ability to conclusively state whether the decrease in longitudinal sound velocity is from partial melting and entering a mixed-phase or a softening of the shear modulus when approaching melt. It is conceivable that bridgmanite has orientation-dependent strength, which can manifest in melting occurring at different shock pressures. This could result in solid bridgmanite pockets within a liquid medium, where the individual solids could possibly support longitudinal and bulk waves (Supplementary Fig. 5). Assuming a mixed phase, the inferred melting temperature would be about 8350 K by interpolating between the solid and liquid Hugoniot temperatures using a Poisson ratio of 0.37 based on the measured *V*_*p*_ and *V*_*B*_ values at 500 GPa (Fig. 2b). This temperature is about 1000 K lower than the upper bound and it brings to a better agreement with the theoretical calculation^28^. The measurement represents a direct detection of melting of silicate at such extreme pressure and it is only possible because the dense silicate starting material allows us to access a cooler Hugoniot *P–T* path. Previous shock experiments with low-density starting materials reach melting below 180 GPa^12,13^.

Constrained by the melting temperatures at low pressure from static laser-heating experiments^11^ and gas-gun shock compression^12^, the upper bound of the MgSiO_3_ melting curve can be expressed by the Simon power law, *T*_*m*_ = 6295(*P*/140)^0.317^. This melting curve is substantially higher than the previously predicted melting curve up to 380 GPa by ab initio two-phase molecular dynamics simulations^28^ and the estimated melting from laser-shock of enstatite^13^ (Fig. 2b). The main uncertainty in the constrained melting temperature at 500 GPa comes from where to place the data along the Hugoniots. The inferred temperature could be 1000 K lower than the upper bound at 500 GPa as discussed above, resulting in a lower melting curve, *T*_*m*_ = 6000(*P*/140)^0.26^. This is still the highest experimentally determined melting. Given the excellent agreement of the calculated Hugoniot densities with the measurements (c.f. Fig. 1), the calculated Hugoniot temperatures should be representative of the true values although the actual temperatures were not directly measured by the experiments. The estimated melting temperature uncertainty from the DFTMD results is ±300 K given a melting pressure uncertainty of ±7 GPa.

### Implications for super-Earth mantles

**Fig. 3:**
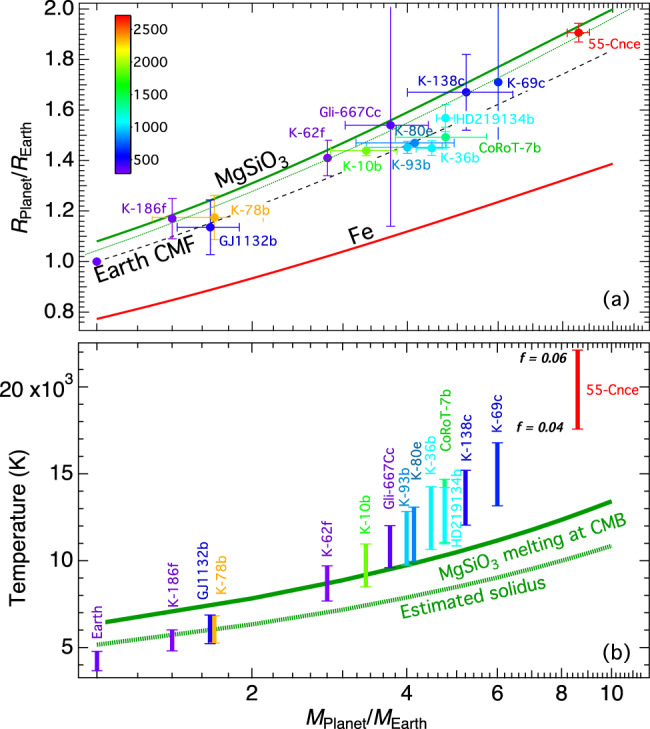
Calculated radius ratios and temperatures at the core-mantle boundary after accretion as a function of mass ratios. **a** MgSiO_3_ mantle is represented by a green solid curve, pure Fe core by red solid curve^30^, and MgSiO_3_–Fe planet with Earth CMF by black dashed curve. The calculated curve for a FeO-free Earth-like mantle^35^ with Mg/Si = 1.07 (green dotted curve) is shown for comparison. Observations for selected super-Earths (www.exoplanet.eu) are shown with a color scale indicating the estimated planetary equilibrium temperatures in Kelvin. Data without horizontal errors indicate that the mass is not well constrained. **b** Colored symbols represent the calculated CMB temperatures of the selected super-Earths, compared with the MgSiO_3_ melting curve and its estimated solidus temperatures calculated by *T*_solidus_ = *T*_melting_/(1 − ln0.79)^37^. The lower bounds were calculated using the Earth-like *f* = 0.04 whereas the upper bounds using *f*  = 0.06.

Accurate determination of the Hugoniot densities for MgSiO_3_ from both experiments and theory provides a strong basis for establishing a reliable thermal equation of state that is applicable to the density profile of the silicate mantle of super-Earths up to at least 8*M*e. For the first time, we obtained high-quality density measurements of solid MgSiO_3_ under extreme conditions that are used as benchmarks for theoretical calculations. By combining the static room-temperature compression data^3–6^ with Hugoniot measurements and the calculated temperatures, we describe the pressure–density–temperature relations of MgSiO_3_ solid by the Vinet equation of state^29^ at 300 K with the thermal pressures calculated from the Mie–Grüneisen relation (see “Methods”), using the optimized parameters: *ρ*_0_ = 4.176 g/cm^3^, *K*_0_ = 265.5 GPa, *K*_0_’ = 4.16, *γ*_0_ = 1.57, *q* = 0.5, and *θ*_*D*_ = 1000 K. With a model MgSiO_3_ solid mantle of super-Earth and a mantle potential temperature of 1725 K, the calculated relation between mass and radius ratios is shown in Fig. 3. The result of an iron core based on recent density measurements of pure iron up to 1400 GPa by laser-driven ramp compression^30^ is also shown. The mass-radius relation for the Earth core/mantle fraction (CMF) is interpolated. The calculation did not include the effect of possible phase transformations beyond post-perovskite because these transformations have a minuscule effect on the mass-radius relation as shown by our recent work^31^.

Our calculated results represent the best-estimated density profile for a model MgSiO_3_ mantle of super-Earths up to 8*M*_E_, with a combination of direct density measurements and ground-truthed DFTMD calculations, because the core–mantle boundary pressure for a super-Earth with 8*M*_E_ is about 1200 GPa. It is important to establish a reference mantle density profile using MgSiO_3_ as an end-member component, as the mantle composition variations can be discussed using a concept of “mantle density excess” defined by the difference between the mantle density and the MgSiO_3_ reference profile, similar to the “core density deficit” for the Earth’s core. For the observed solar Fe, Mg, and Si abundances^14,15^, differentiated rocky planets would leave a silicate mantle enriched in iron although the amounts of Fe enrichment depend on the bulk iron content and the oxidation state during accretion. The mantle density excess comparing to a MgSiO_3_ reference would be a quantitative measure of the Fe-enrichment. Similarly, a silicate mantle with Mg/Si > 1 would lead to a denser mantle due to highly compressible MgO and the transition of MgO to a denser CsCl structure in the deep mantle^32^. Strongly preferred Fe partitioning into the oxide would further increase the density. However, the Silica-saturated mantle could have the opposite effect on density because high-pressure SiO_2_ phases are relatively incompressible^33^, even with the formation of the denser high-pressure pyrite-type phase^34^.

The well-defined reference density model is a useful tool to track the chemical variations within the mantle. The mass-radius ratio relation of the MgSiO_3_ mantle is also critical for understanding the core–mantle fraction and the presence or proportion of the H_2_O or gas component in super-Earths. Our calculated mass-radius curve (Fig. 3) shifts up comparing to the commonly cited curve for MgSiO_3_^35^ which significantly overestimated mantle density based on large extrapolation and theoretical calculations without benchmarks of experimental data. The difference could lead to different interpretations of the observations. For example, exoplanet *55 Cnc e* with a radius ratio of ~1.9, has a high planetary equilibrium temperature (>2000 K), and it plots at the different sides of the model mantle curve (Fig. 3). Planets with radius ratios above ~1.5 generally have thick volatile envelopes, but *55 Cnc e* may have suffered substantial atmospheric loss given its close, hot orbit. Being above the curve (dotted green) implies that the planet has to include light components such as H_2_O which has to reconcile with the high planetary temperature according to the previous model. Our result indicates that the observation could be simply interpreted by core-mantle fraction because it plots below our calculated curve (solid green). With the ideal end-member mantle and core compositions, the estimated CMF is about 6% and the density profile of the interior is illustrated in Supplementary Figure 6. Such a small iron core would indicate a very low Fe/Si ratio (~0.32), lower than that of any known chondrites. Of course, there are compositional degeneracy and large observational uncertainty to be considered for a detailed model, but it illustrates the importance for an accurate reference mantle model because a different first-order question might be emphasized. Similarly, several relatively well-characterized “hot” super-Earths (e.g., Kepler 10b, 36b, 80e, and 93b, CoRoT-7b, and HD-219134b) plot close to the Earth CMF curve clustered near 4*M*_*E*_ (Fig. 3), implying a similar metal-silicate ratio as the Earth without considering the trade-off between mantle composition and CMF. On the other hand, the “cold” super-Earths (e.g., Kepler 186f, 62f, and 138c, and Gliese-667Cc) plotted close to the MgSiO_3_ curve, may need to add an H_2_O component in order to have a sizable iron core. Although large uncertainties in interpretation will remain without the improved mass and radius measurements and additional observations, the accurate density measurements of MgSiO_3_ mantle from this study and Fe core by laser-driven compression^30^ represent a significant advance from mineral physics towards interior models of exoplanets.

Understanding the melting temperature in the deep mantle is important for the mantle dynamics and evolution of super-Earths. The observed high melting temperature of MgSiO_3_ at great depths provides an upper bound for modeling deep mantle melting processes. It requires more energy to be retained from accretion to melt the mantle, therefore, influence the depth of the magma ocean if global melting occurs at the end of planetary accretion. We recently investigated how the initial internal heat of the planet after formation scales with planet size^31^. Figure 3b shows the calculated initial temperatures at the core–mantle boundary (CMB) following accretion of the super-Earths, assuming simple MgSiO_3_-mantle and Fe-core. Depending on the heat retention efficiency (*f*) during accretion, the calculated CMB temperatures of the super-Earths (*M*_*E*_ > 2) with an Earth-like *f* = 0.04 or higher, are sufficient to melt the entire mantle even with our high-MgSiO_3_ melting temperature (Fig. 3b). The calculation is based on a mantle potential temperature of 1725 K^36^. For a higher mantle potential temperature in the early history, it is expected that extensive mantle melting in super-Earths would be common following accretion. A similar conclusion was reached in a recent thermal model of super-Earths^37^.

The high melting temperature of MgSiO_3_ at 500 GPa implies a high mantle solidus in super-Earths. If the mantle temperature is anywhere near its solidus, the core would be fully liquid and thermal convection or supersaturation of light elements in the liquid core such as MgO or SiO_2_ is required to drive the dynamo^38–40^. Once the mantle and core cool down enough for the core to start to solidify, the mantle temperature would be very far below its solidus because the mantle solidus is a stronger function of planet mass than the mantle adiabat (Fig. 2b). Therefore, the mantles of massive planets could be more viscous, resulted from the high melting temperature, which would reduce the core cooling rate. Earth’s core is thought to be marginal for thermal convection today (i.e. the CMB heat flow is similar to the conductive requirement to drive convection)^41^. Compared to Earth, therefore, the cores of more massive planets tend to cool more slowly due to the increased mantle viscosity and have a higher core thermal conductivity, implying that thermal convection is less likely. Compositional convection in the core would also be less likely in massive planets because their cores initially start higher above the core liquidus^31^ and a lot more cooling is required to begin core solidification, compared to Earth. In conclusion, massive rocky planets may be able to drive a dynamo early on when cooling is fast, then have no dynamo for billions of years while the interior is slowly cooling, and then possibly drive a compositional dynamo billions of years later.

## Methods

### Experiments

The dynamic compression experiments were performed at the Sandia National Laboratory Z machine^42^ that is capable of generating magnetically-accelerating flyer plates with impact velocities up to 42 km/s. We used solid aluminum or composite copper/aluminum flyer plates as impactors for the plate-impact experiments. The copper/aluminum flyer plate consists of a nominally 250-micron copper layer supported by a 0.9 mm aluminum layer precisely fabricated by diamond-turning^24,26^. The target was assembled in the coaxial load geometry, consisting of a north and a south sample panel with seven sample holders on each panel (Supplementary Fig. 1). The pre-synthesized polycrystalline MgSiO_3_-bridgmanite samples were nominally 2.3-mm disks with variable thicknesses (0.3659–0.9339 mm), backed by Z-cut α-quartz windows. The bridgmanite samples are not transparent. Adjacent samples were transparent quartz or diamond/quartz windows which enabled observation of the flyer plate and determination of the impact time at the sample.

Syntheses of the MgSiO_3_-bridgmanite samples were carried out in the multi-anvil press at the Carnegie Institution for Science^43^. The high-pressure assembly used in this study is similar to that of the previous study^9^, but the tungsten–carbide cubes were replaced by 25.4-mm sintered-diamond cubes. Cylindrical bridgmanite samples with diameters of ~2.3-mm were synthesized at 25 GPa and 1673 K, using the same sintered MgSiO_3_ enstatite as starting material for all the syntheses. The recovered samples were polished into disks with parallel surfaces. More than 34 bridgmanite samples were prepared for the shock wave experiments. The chemical compositions of the recovered bridgmanite were confirmed by electron microprobe analyses, with the representative composition of MgO (40.70 ± 0.39 wt%) and SiO_2_ (59.85 ± 0.31 wt%), consistent with the MgSiO_3_ chemical formula. The density of the crystal is 4.103 ± 0.002 g/cm^3^, determined from its unit cell parameters (*a* = 4.7763 ± 0.0006 Å, *b* = 4.9314 ± 0.0006 Å, and *c* = 6.8992 ± 0.0010 Å) by X-ray diffraction technique. Densities of selected samples were also measured by the Archimedes method, with an average value close to 4.10 g/cm^3^, but the uncertainty is relatively large (~1%) because of the small sample size.

For symmetric-target plate-impact experiments, the north and south flyer plates impacted the corresponding targets (the north and south sample panels) at the same velocity (Supplementary Fig. 1). The flyers were launched at impact velocities between 9.5 and 30.0 km/s (Table 1). For the two highest pressure experiments (Z3250 and Z3321), the impact velocities were intentionally slightly different on the north and south panels, and the data were analyzed separately for the two panels. The flyer velocity and impact time at the sample was measured through adjacent transparent windows by multifiber VISAR probes^25^. Corrections are applied for dynamic flyer tilt (interpolation) and measured flight gap differences from sample to sample due to imperfect assembly/mounting of the target panel. The measured impact time difference from the top to the bottom of the panel is typically ~1–3 ns. Part of this is due to dynamic flyer bowing/tilt during acceleration and part due to variations in the flight gap distance between the initial flyer position and the samples (assembly and alignment precision). The flight gap correction is typically on the order of ~0.5 ns (resulting from ~10-micron differences in flight gap). These corrections are small, but important, and were applied for every bridgmanite Hugoniot measurement presented using the unique above/below-measured impact times and flight gaps.

Additional velocity histories were recorded in 3 channels for each bridgmanite sample with three different fringe sensitivities (typically 405, 1057, 2290 m/s/fringe in a vacuum). The bridgmanite shock velocity (*U*_*s*_) was determined from the transit time for the shock to traverse the sample. The uncertainties in shock velocity are less than 2%. The bridgmanite particle velocity (*u*_*p*_) and associated uncertainty were determined with impedance matching in a Monte Carlo technique which varied the equation of state of the flyer by ±3% and the flyer velocity, bridgmanite shock velocity, and bridgmanite initial density within the stated uncertainties; the mean and standard deviation of the particle velocity are reported in Table 1. The copper equation of state used was SESAME 3325, and the aluminum equation of state used was SESAME 3700^44^. The Hugoniot in both the copper and aluminum equation of state models utilized for impedance matching the bridgmanite Hugoniot have been validated by multiple studies^24,45^. For several experiments (e.g., Z3011, Z3029, and Z3116), copper and aluminum samples were also mounted as standards for additional validation.

### Sound velocity analysis

The sound velocity was determined using the overtaking rarefaction method with quartz windows. In the overtaking rarefaction method, the backward-facing shock in the composite flyer reaches the Cu/Al interface after propagating a known distance. Upon reaching the interface, the shock pressure releases due to the large density discontinuity, resulting in a rarefaction wave that propagates through the flyer and sample, eventually overtaking the shock front. This method has previously been used to measure the sound velocity in shocked copper^24^ and beryllium^26^. The use of quartz windows decreases the uncertainty in the overtaking time and enables additional analysis of the wave profile. For all shots, the shock transmitted from the Bridgmanite into the quartz window was of sufficient strength to melt and partially ionize the quartz, generating a reflecting shock front which was observed with VISAR. Upon overtake of the rarefaction wave, the shock velocity begins to decrease and is clearly visible in the wave profile.

The bridgmanite sound velocity was analyzed using both multi-sample and single-sample techniques to determine the overtake time and thickness. Because the single-sample technique requires an assumption of a scaled sound velocity for waves propagating through released material^22^, this technique was used for the shots where velocity could not be determined by the multi-sample method. In both techniques, the wave profile was discretized using linear fits to the constant velocity plateau, longitudinal release (if present), and bulk release (Supplementary Fig. 4). The plateau fit and release fits were extrapolated to find their intersection, which determined the overtake time in the window for a particular sample.

The multi-sample technique leveraged the different bridgmanite sample thicknesses fielded to relate the window overtake time (*t*_WO_) with the sample thickness. The sample thickness was linearly fit as a function of *t*_WO_ and extrapolated to *t*_WO_ = 0. This determined the depth (*d*_*∞*_) and time (*t*_*∞*_) at which overtake would occur in an infinitely thick bridgmanite sample. The bridgmanite sound velocity is given as

1 $$C_L = \frac{{d_\infty }}{{t_\infty - t_L}}$$

where $t_L = t_I + \frac{{d_f}}{{U_S^{Cu}}} + \frac{{d_f}}{{C_L^{Cu}}}$ is the time when the rarefaction wave enters the bridgmanite, *t*_*I*_ is the impact time, *d*_*f*_ is the thickness of the copper layer on the flyer, and $U_S^{Cu}$ and $C_L^{Cu}$ are the copper shock velocity and Lagrangian sound velocity, respectively. This technique was used to determine the bulk wave speed in both solid and liquid bridgmanite.

The longitudinal wave speeds in solid bridgmanite were determined by the single-sample analysis. The wave profiles from two low-pressure shots showed release from a solid-state (Supplementary Figs. 4b, c). In the single-sample technique, the overtaking wave propagates through both shocked and partially released bridgmanite prior to breaking out into the quartz window. The amount of shocked and partially-released material that the overtaking wave traverses is determined by solving the system of equations that define the bridgmanite characteristics shown in Supplementary Fig. 3. The three waves which impact the overtake are the overtaking and window rarefactions (dashed red lines) and partially-released rarefaction (dashed-dotted purple line). This system defines the characteristic intersection (*x*_int_, *t*_int_) and Lagrangian sound velocity as

2 $$t_{\rm{int}} = \frac{{S_{C_L}t_{\rm{IO}} + t_q}}{{1 + S_{C_L}}}$$

3 $$C_L = \frac{{d_b}}{{2t_{\rm{int}} - \left( {t_q + t_L} \right)}}$$

4 $$x_{\rm{int}} = C_L\left( {t_{\rm{int}} - t_L} \right)$$

where $S_{C_L}$ is the sound velocity scale factor for partially released bridgmanite, *t*_IO_ is the overtake time at the bridgmanite–quartz interface, *t*_*q*_ is the time of shock breakout into the quartz window, and *d*_*b*_ is the bridgmanite sample thickness.

Single-sample analysis of the three shots where the wave profiles exhibited a simple release determined $S_{C_L}$ = 0.89 ± 0.07. Copper and quartz simulations^24,26^ have previously indicated that the scale factor is approximately pressure-independent so the constant value remains valid for the low-pressure data even though it was calculated from the three highest-pressure shots. The overtake time was determined by using a characteristics method to transform the quartz shock velocity into an interface particle velocity^46^ and fitting the plateau and release regions of the particle velocity. This method used the most recent quartz Hugoniot and release models^47^ to define the quartz sound velocity as a function of the measured shock velocity.

Because the single-sample analysis only considers first-order characteristic interactions, it is only valid for the initial release wave and cannot be used to determine the bulk sound speed in a solid. Extension to the bulk speed would require additional scale factors for second-order interactions that are unable to be constrained experimentally.

On shot Z3203, the wave profile indicates that a three-stage release occurred (Supplementary Fig. 4c), with the initial release attributed to an edge rarefaction impinging on the shock front in the center of the target prior to overtaking of the longitudinal wave. The edge wave was also observed in the shot Z3029 (Supplementary Fig. 5). As the bridgmanite samples were irregularly shaped, the edge wave could not be used for sound speed analysis, and modification to the overtaking wave analysis was required. From the behavior of the shock front and overtaking rarefaction in the quartz window shown in Supplementary Fig. 3, the time at which the rarefaction overtakes the edge wave accurately represents the overtake that would occur without an edge wave present (Δ*t* < 0.1 ns). Fitting to the plateau generates an overtake time ~1.5 ns earlier, significantly increasing the measured sound velocity. Because the edge wave propagates inward with nominally constant velocity, the expected time where the edge wave reaches the center of the bridgmanite/quartz interface is approximately the same as when it reaches the center of the shock front. To account for this, the quartz window analysis was modified such that at each time-step, the characteristic sound velocity was adjusted according to the edge wave pressure at the shock front. This analysis is unable to account for edge waves propagating at the longitudinal velocity in the bridgmanite impacting the overtaking wave, and hence should be considered an upper bound on the sound speed. Similarly, the bulk velocity calculated with the multi-sample analysis should be considered an upper bound due to unaccounted edge wave effects.

Uncertainties were calculated using a Monte Carlo method with 10^5^ iterations. For the overtake times, plateau and release regions of interest were defined and the fit was calculated with variable-length intervals of 10–15 ns for the plateau and 5–10 ns for the release. For experiments where a release region was shorter than 10 ns the fit interval range was 0.5–1× the region of interest. Typical uncertainties in overtake times were <2 ns, and the overtake transit time was the dominant uncertainty for the bulk of these experiments. The thickness of the copper layer on the flyer was calculated from the copper and aluminum density and measurements of the flyer mass and dimensions. The overall flyer thickness was measured to <0.5 µm using an enhanced dual confocal microscope^48^, and lateral dimensions and mass uncertainties were ~10 µm and ~0.1 mg, respectively. This produced a copper thickness uncertainty of ~1 µm. On Z3203, accounting for the edge wave introduced a systematic error of ~3.5% to both the longitudinal and bulk sound speed; this produced total uncertainties of ~11% and ~13%, respectively.

### Computational methods

We performed a series of DFT calculations for the bridgmanite, post-perovskite, and liquid phases of MgSiO_3_. All calculations were performed using the VASP version 5.4 code, implementation of Kohn–Sham DFT using a plane-wave basis, projector-augmented-wave pseudopotentials, and periodic boundary conditions^49–51^. The exchange and correlation contributions to the energy were computed using the Perdew, Burke, Ernzerhof generalized gradient approximation^52^. The VASP projector augmented wave pseudopotentials were used with a plane wave energy cutoff of 800 eV to ensure convergence of the stress tensor. To accurately capture the semi core excitations in magnesium at high temperatures, we used a magnesium pseudopotential with a 2*p*^6^3*s*^2^ valence configuration, while that of silicon and oxygen were 3*s*^2^3*p*^2^ and 2*s*^2^2*p*^4^, respectively. All calculations used 160 atom supercells for the bridgmanite, post-perovskite, and liquid phases.

First, the equations of state (EOS) for the static lattice were obtained for bridgmanite and post-perovskite from a Vinet EOS^29^ fit the total energy of the lattice computed for a series of densities. A 6 × 6 × 6 k-point grid was found to yield converged values of the energy and stress tensor. For each density, the crystal geometry was optimized until the average force per atom was less than 0.01 meV/atom, but the overall crystal symmetry was constrained to be orthorhombic.

Second, the principal Hugoniot of bridgmanite was computed from a set of DFTMD calculations. Our DFTMD calculations follow a similar approach as in previous work^17,53^. The Brillouin zone was sampled at a single *k*. The electronic occupations were populated according to the Mermin finite temperature formulation of DFT^54^. The reference state for the Hugoniot calculations was obtained from the energy and pressure of a well-equilibrated DFTMD calculation of bridgmanite at 300 K with a density of 4.10 g/cm^3^. Hugoniot states were then computed from linear interpolation of the temperature dependence of the energy and pressure for a set of isochores such that the Rankine–Hugoniot relation was satisfied

5 $$E - E_0 = \frac{1}{2}\left( {P + P_0} \right)\left( {V - V_0} \right)$$

With *E*, *P*, *V* denoting the energy, pressure, and volume of the Hugoniot state, respectively, and the subscript indicating values for the reference state of bridgmanite. For bridgmanite and post-perovskite, the lattice parameter ratios for the high-temperature DFTMD calculations were unchanged from their optimized 0 K values. Results of the DFTMD calculations for MgSiO_3_ post-perovskite and the liquid phase are shown in Fig. 1.

With the Hugoniot states in hand, we then computed the bulk sound velocity in the liquid phase. For a point on the Hugoniot, the adiabatic bulk modulus is given by

6 $$K_S = \left( {P - P_0} \right)\frac{\gamma }{2} + \rho \left. {\left( {\frac{{dP}}{{d\rho }}} \right)} \right|_H\left[ {1 + \left( {1 - \frac{\rho }{{\rho _0}}} \right)\frac{\gamma }{2}} \right]$$

With $K_S$ the adiabatic bulk modulus, $P - P_0$ the pressure difference between the Hugoniot and reference state, $\gamma$ the Grüneisen parameter, $\rho$ the density on the Hugoniot, and $\left. {\left( {\frac{{dP}}{{d\rho }}} \right)} \right|_H$the derivative of the Hugoniot pressure with respect to the Hugoniot density, evaluated at the point on the Hugoniot in question. The only term which requires some care is the estimation of the pressure derivative. A typical Hugoniot calculation is done on a coarse density grid, which makes a finite difference estimation of the derivative suspect. We found that fitting the entire *P,ρ* Hugoniot curves with a cubic polynomial to provide a more robust, and analytic, estimate of this term. We call this the “derivative method”.

The resulting estimates of the bulk modulus were cross-checked by performing a set of follow-up calculations. It is well-known that compression of a hydrodynamic material due to an infinitely weak shock is isentropic. Therefore, the isentropic bulk modulus can be estimated directly according to the definition

7 $$K_S = \rho \left( {\frac{{\partial P}}{{\partial \rho }}} \right)_S \cong \frac{{P_2 - P_1}}{{1 - \frac{{\rho _1}}{{\rho _2}}}}$$

where a pair of points (*P*_1_,*ρ*_1_ and (*P*_2_,*ρ*_2_) provide that the latter point is on the Hugoniot for which the former point is the reference state. In other words, taking (*P*_1_, *ρ*_1_) as a reference state, one satisfies the Rankine–Hugoniot relations for a density *ρ*_2_ a small distance away from *ρ*_1_. We call this the “release method” because in the experimental literature such states are sometimes called *release* states, that describe isentropic decompression of a material after the shock wave has passed through. The results from the “release method” are consistent with those from the “derivative method”. The bulk sound velocities calculated from the bulk modulus and density as a function of pressure are listed in Supplementary Table 1. We also provided the statistical uncertainties in the Hugoniot states which give the statistical uncertainties in the underlying *E*(*V, T*), and *P*(*V, T*) data from the DFTMD calculations. The small errors in pressure indicate that the underlying data is well equilibrated and therefore representative of the system in equilibrium. Comparison of the experimental and theoretical estimates are shown in the main text in Fig. 2a.

### Thermal equation of state

The pressure(*P*)–density(*ρ*)–temperature(*T*) relation is calculated by

8 $$P(\rho ,T) = P(\rho ,300K) + P_{th}$$

where *P*(*ρ*,300 K) is calculated by the Vinet equation

9 $${\it{P}}\left( {\rho ,300{\mathrm{K}}} \right) = 3{\it{K}}_0\left( {\frac{{\rho _0}}{\rho }} \right)^{ {\!}- 2/3}\left[ {1 - \left( {\frac{{\rho _0}}{\rho }} \right)^{1/3}} \right]{\mathrm{exp}}\left\{ {\frac{3}{2}\left( {{\it{K}}_0^\prime - 1} \right)\left[ {1 - \left( {\frac{{\rho _0}}{\rho }} \right)^{1/3}} \right]} \right\}$$

The thermal pressure (*P*_th_) is expressed by the Mie–Grüneisen relation

10 $$P_{th} = \frac{\gamma }{V}\left[ {E\left( {T,\theta _D} \right) - E\left( {300{\rm{K}},\theta _D} \right)} \right]$$

with the Grüneisen parameter, *γ* = *γ*_0_(*ρ*_0_/*ρ*)^*q*^, and the Debye temperature, *θ*_*D*_ = *θ*_*D*0_exp[(*γ*_0_ − *γ*)/*q*]. The harmonic internal energy *E*(*T*, *θ*_*D*_) is calculated from the Debye model^55^.

The thermal equation of state with the optimized parameters reproduces the static room-temperature compression data^4–6^ and the Hugoniot measurements up to 500 GPa and 9000 K (Fig. 1). Using these new parameters, we calculated the internal structure of super-Earths up to 10*M*_*E*_, following the same procedure described in our recent paper^31^. Supplementary Fig. 6 shows representative density profiles super-Earth with 4*M*_*E*_ and the exoplanet 55 Cnc e, with a mantle potential temperature of 1725 K.

In order to estimate the initial temperatures at the CMB following super-Earth accretion, we calculated temperature as a function of mass from the accretional heat and compression heat^31^. Supplementary Fig. 7 shows the contribution of the gravitational energy that is retained from accretion, ∆*T*_G_, with an efficiency factor *f* = 0.06 and an Earth-like *f* = 0.04, and the mantle adiabat, ∆*T*_*ad*_, with a mantle potential temperature of 1725 K. The ∆*T*_*G*_ has a stronger mass dependence, comparable to the previous model^37^. The mantle potential temperature could be much higher immediately after accretion that would make the global magma ocean in super-Earths common.

## Supplementary information

Supplementary Information

Peer Review File

## Data Availability

All data generated or analyzed during this study are included in this published article and its Supplementary Information files.
