# Supplementary material for: Melting and density of MgSiO3 determined by shock compression of bridgmanite to 1254GPa
Source: Nat Commun. 2021 Feb 9;12:876. doi: 10.1038/s41467-021-21170-y (PMC7873221; doi:10.1038/s41467-021-21170-y)
Supplement: Supplementary file 1 — Supplementary Information [file 41467_2021_21170_MOESM1_ESM.docx]

**SUPPLEMENTARY INFORMATION**

**Melting and density of MgSiO_3_ determined by shock compression of bridgmanite to 1254 GPa**

Yingwei Fei^1,*^, Christopher T. Seagle^2^, Joshua P. Townsend^2^, Chad A. McCoy^2^, Asmaa Boujibar^1^, Peter Driscoll^1^, Luke Shulenburger^2^, Michael D. Furnish^2^

^1^Earth and Planets Laboratory, Carnegie Institution for Science, 5251 Broad Branch Road, NW, Washington, DC 20015, USA

^2^Sandia National Laboratories, Albuquerque, NM 87185, USA

^*^Correspondence to: Yingwei Fei ([yfei@carnegiescience.edu](mailto:yfei@carnegiescience.edu))

Supplementary Table 1

Supplementary Figures 1-7

**Supplementary Table 1.** Calculated Hugoniot data, bulk modulus, and bulk sound velocity.

Note: Bridgmanite reference state for the calculations: *E*_0_ = -33.072 MJ/kg, *ρ*_0_ = 4.10 g/cc, *P*_0_ = 10.302 GPa, and *T*_0_ = 299.977 K


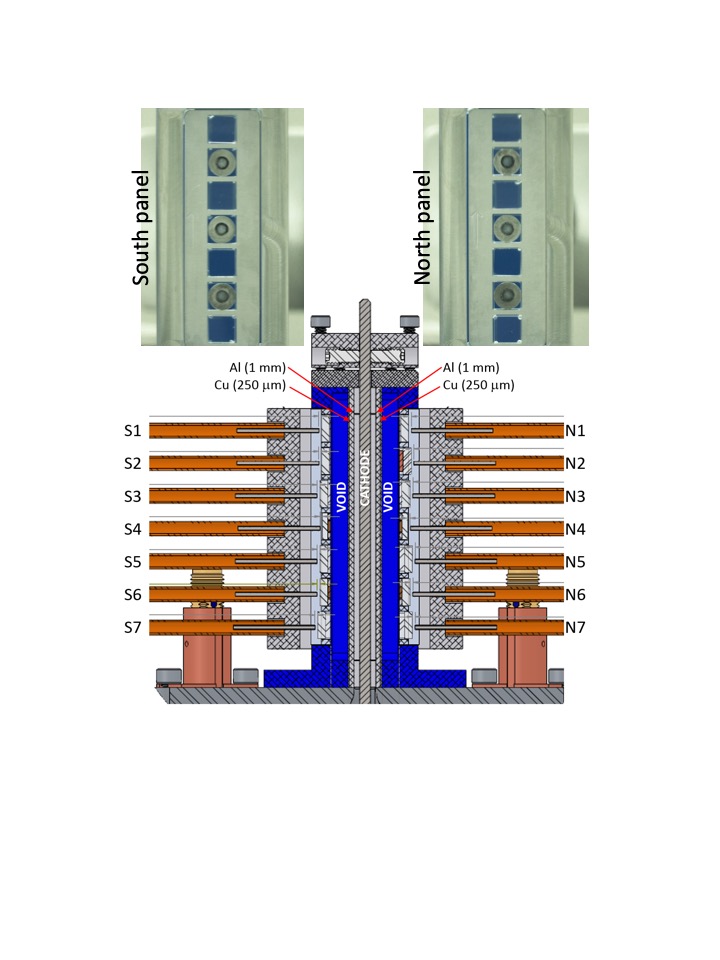


Supplementary Fig. 1. **Representative target configuration (Z3250) of the coaxial load geometry.** Labels S1 – S7 and N1 – N7 correspond to the VISAR probes as well as to the samples monitored. The bridgmanite samples are located at S2, S4, and S6 on the south panel and at N2, N4, and N6 on the north panel. The Cu/Al flyer separated from the cathode stalk at the center by A-K gaps (anode-cathode gap distances) in both sides. When firing the Z machine, the huge magnetic field in the A-K gaps generated by the stored energy from the capacitor banks drives the flyers to impact the target.

Supplementary Fig. 2. **Shock velocity *U_S_* versus particle velocity *u_p_* for MgSIO_3_-bridgmanite starting material.** Data from this study (purple circles) are compared with liquid MgSiO_3_ data by the laser-driven shock (red open squares)^18^, and solid MgSiO_3_ data (black solid diamonds^9^ and open diamonds^10^) by gas-gun method. The purple solid and dashed lines are the calculated results for post-perovskite and liquid, respectively, by DFTMD simulations.


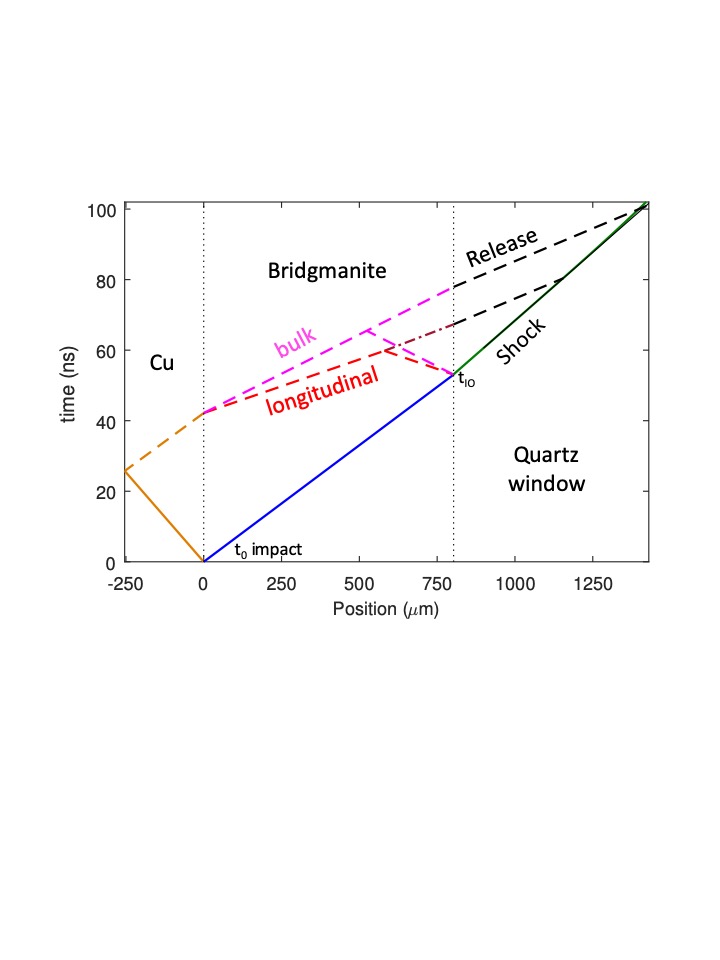


Supplementary Fig. 3. **Wave interactions in *x-t* diagram for overtaking wave analysis in solid.** The bridgmanite sound velocity was determined from the overtake time of rarefaction waves (dashed lines) with the shock front (solid lines). When the rarefaction wave from the copper/aluminum interface (orange) reaches the bridgmanite interface, it splits into longitudinal (red) and bulk (pink) overtaking waves. These waves are transmitted into the quartz window (black) where the overtake the quartz shock front (green). In the single-sample analysis, the overtaking wave interacts with a rarefaction wave from the bridgmanite/quartz interface and propagates at a reduced sound velocity (dashed-dotted purple line) which is incorporated into the analysis. The edge wave in the quartz window decreased the shock velocity such that the true overtake time is later than that at the edge wave by ~0.1 ns and ~1.5 ns for the longitudinal and bulk waves, respectively. Note that no interactions between the longitudinal and bulk waves in the bridgmanite were accounted for, so the extracted sound velocity should be considered an upper bound.

Supplementary Fig. 4. **Representative wave profiles for bridgmanite shocked into the liquid (a) and solid (b and c) phases.** **(a)** In the liquid phase, the bridgmanite shock velocity (solid blue line) exhibits a single-stage release that can be fit as a constant-velocity plateau (dashed black line) and bulk release (dashed-dotted green line). The intersection of these fits determines the overtake time for a given sample. **(b)** Shots with peak pressures below melting show a longitudinal release (dashed-dotted purple line) followed by a bulk release (dashed-dotted green line). **(c)** For the shot Z3203, an edge wave (dashed red line) reached the center of the target prior to the rarefaction wave overtaking the shock front, in addition to the longitudinal (dashed-dotted purple line) and bulk release (dashed-dotted green line).

Supplementary Fig. 5. **Wave profiles for shot Z3029 at 500 GPa.** **(a)** Wave profiles from two samples on the north panel show an edge wave followed by a longitudinal wave. One of the samples also show a bulk release wave that allow us to estimate the bulk sound velocity. **(b)** Wave profiles from two samples on the north panel show the edge and longitudinal waves.

Supplementary Fig. 6. **Calculated density profiles for exoplanet 55 Cnc e (red) and super-Earth with 4 *M_E_* (blue).** The methods for calculating internal structure were described in publication^32^. The first density jump is caused by the transition to bridgmanite. There is a small density jump (~1%) at depth ~1200 Km, caused by the transition to post-perovskite. The post-perovskite mantle density profile is calculated using the parameters optimized in this study. The core density is derived from that of pure iron^30^.

Supplementary Fig. 7. **Calculated initial temperatures at the core-mantle boundary (*T*_CMB_) after accretion as a function of mass, with an efficiency factor *f* = 0.06.** The temperature contribution of the gravitational energy (∆*T*_G_) and mantle adiabatic compression (∆*T*_ad_) with a mantle potential temperature of 1725 K) are shown. The calculated ∆*T*_G_ with an Earth-like *f* = 0.04 is compared with model of Stixrude^33^. The MgSiO_3_ melting temperatures of this study are also compared with previous extrapolations^33^.
