# Supplementary material for: Melting and density of MgSiO3 determined by shock compression of bridgmanite to 1254GPa
Source: Nat Commun. 2021 Feb 9;12:876. doi: 10.1038/s41467-021-21170-y (PMC7873221; doi:10.1038/s41467-021-21170-y)
Supplement: Supplementary file 2 — Peer Review File [file 41467_2021_21170_MOESM2_ESM.pdf]

Reviewer #1 (Remarks to the Author):

Bridgmanite is the most abundant mineral in Earth's silicate mantle and is almost certainly an important constituent of rocky exoplanets. The melting curve of bridgmanite at extremely high temperatures is critical to models of planetary structure and evolution. However, experiments that directly measure melting temperatures at extreme conditions are difficult and scarce. This compelling, well-written manuscript reports new data from shockwave loading of pre-synthesized bridgmanite. The melting temperature at very high pressures is higher than expected based on prior studies, which could have important implications for the likelihood that accretional heating melts the mantle and the subsequent vigor of mantle convection.

I have one concern about the interpretation of the experimental data, which I think stems from my inexperience with actually performing shock experiments. Hopefully, addressing this concern will make the manuscript more digestible for a broader audience. I also have a few minor comments about the general framing of the study. Assuming the authors address these comments, I enthusiastically recommend publication.

Moderate comment:

As a non-practitioner of shock experiments, I was confused about some of the implications of using "dense $MgSiO_3$" in the experiments. I suggest quoting the actual pressure and temperature conditions at which the samples were synthesized in the main text. Currently, "lower-mantle pressures" are mentioned in page 2, but it would be worth quoting 25 GPa and 1673 K (as noted in the Methods). Basically, I would clarify for the general reader that the experimental samples are ~33% denser than usual because they have already been subjected to inelastic compression, and not because of porosity or compositional effects.

More importantly, I think the methods description on pages 16 and 17 is a bit incomplete. Specifically, what is the reference pressure (P_0) used in Eq. 5 (Rankine-Hugoniot relation) and the following equations? Page 16 states that it "was obtained from the energy and pressure of a well-equilibrated DFTMD calculation of bridgmanite at 300 K with a density of 4.1 g/cc." However, I did not see the reference pressure explicitly quoted in the text, Table S1, Figure 1. I *assume* that reference pressure close to the synthesis pressure (25 GPa)? I presume that "dense $MgSiO_3$" has a higher (?) melting temperature at ambient pressure than regular $MgSiO_3$ with a density of ~3.2 g/cc. So, if ambient pressure was used as a reference pressure, then the melting temperatures and densities of real bridgmanite in planetary mantles would be over(?)estimated?

Minor comments:
- On page 2, the last part of the last sentence of the first full paragraph ("no direct detection of melting and density…") is incomplete, missing a verb at least.
- On page 6, the last full paragraph states "Given the excellent agreement of the calculated Hugoniot densities with the measurements, we place high confidence of [sic] the calculated Hugoniot temperatures." Please quantify "excellent" and "high confidence."
- On page 8, this discussion should probably note that planets with radii above ~1.5 Earth-radii generally have thick volatile envelopes. 55 Cancri e is a bit of a special case since may have suffered substantial atmospheric loss given its close, hot orbit.
- On page 9, I don't think "homologous law" is a common term in Earth and planetary science. Please add a citation/elaboration.
- On page 9, I think you should relatedly emphasize that this study does not directly compute (correct?) the viscosity of $MgSiO_3$ in super-Earths. The possible temperature-dependence is described, but there also could be pressure effects expressed as a non-zero activation volume (correct?). I think the speculation about mantle dynamics is reasonable, but there are still lots of uncertainties about viscosities within massive planets.

Reviewer #2 (Remarks to the Author):

The paper “Melting and density of $MgSiO_3$ determined by shock compression of bridgmanite to 1254 GPa” is a deep and complex paper that goes beyond the “simple” shock compression experiment as suggested by the title. From sample preparation to shock experiments, DFTMD simulations and a long discussion about possible implications for our knowledge of the internal structure and dynamics of super-Earths, it is obvious that a lot of care has been into every single part of the work presented here. Despite being rich and complex, the paper is for the most part rather easy to read, except perhaps the discussion that is difficult to fully understand without a careful read of a previous paper by the same team (“Super-Earth Internal Structures and Initial Thermal States” by Boujibar et al. 2020). This paper definitely presents brand new results that will be of major interest to a wide audience. My recommendation is to publish it after minor revisions.

The shock experiments give very interesting results and the data quality is very high. There are however two limitations that are not discussed:

- absence of structural measurements: the evolution of the structure of the bridgmanite to ppv and perhaps other phases (a point that is discussed in length in Boujibar et al. 2020 but not here, we’ll see that later) cannot be tracked with this technique (“Our experiments are expected to have shocked the sample into the stability field of post-perovskite and then into liquid state along the Hugoniot.” p. 4). Data fits the lowest P data and are in agreement with simulations that’s right but nonetheless there is no direct evidence that the ppv phase is the stable phase up to 500 GPa and ~ 9000 K. Moreover, the ppv phase is known for its strong anisotropic elastic and rheological properties. What happens during a strong uniaxial shock event? For example, can we expect some preferential orientation of the ppv? As this can influence the measurements done by the authors it would have been interesting to see this issue discussed.

- absence of T measurements. All T reported here are calculated. On p. 6 the authors' claim about the accuracy of the estimation appears however reasonable. P. 6 is pretty critical because it is mostly about the onset of melting recorded at 500 GPa, a very important measurement. That’s a pity that T couldn’t be measured with a streak camera. Looking at Figure S1 it seems pretty difficult to have an optical access to at least one sample, but one can hope for the future. A brief comment about why it couldn’t be done would be interesting.

The discussion about super-Earths internal structure, “mantle density excess”, core solidification and deep mantle viscosity is an interesting read, though difficult to fully follow without carefully reading Boujibar et al. 2020. Three comments:

- H_2O content of exoplanet 55-Cnce p. 8: I understand the exercise here but the uncertainties go far beyond the error bars of Fig. 3a. Perhaps a bit more careful phrasing should be used.

- where does the mantle potential T value of 1725 K come from?

- extrapolation of $MgSiO_3$ melting curve beyond the probable stability field of $MgSiO_3$ ppv. The phase transition from ppv to post-ppv phases is not discussed in this paper but it is in Boujibar et al. 2020. In the P range considered (up to 1.2 TPa) several scenarios are possible. Perhaps this has no effect on the shape of the melting curve, perhaps there are no liquid-liquid transitions to be expected. But I think this could be clarified by the authors.

Reviewer #3 (Remarks to the Author):

Summary:

In this manuscript, Fei et al. investigate the equation of state and melting of $MgSiO_3$ between 296 and 1254 GPa. They report plate-impact shock-compression experiments on pre-synthetized bridgmanite samples carried out at Sandia National Laboratory Z machine.

The pressure and density along the bridgmanite Hugoniot are calculated from the measured mean shock velocity (Us) and flyer plate velocity (Uf) based on impedance matching procedures and Rankine-Hugoniot relations. Moreover, longitudinal and bulk sound velocity measurements are performed using the overtaking rarefaction approach. The absence of longitudinal sound velocity above 500 GPa leads the authors to conclude that melting occurred at 500 GPa along the Hugoniot.

These experimental results are compared to new first-principles atomistic simulations (DFT-MD) of bridgmanite, post-perovskite and liquid $MgSiO_3$. Based on the good agreement between the experimental and numerical results (Hugoniot in the pressure-density space and bulk sound velocity), the authors used the DFT-MD Hugoniot temperatures to estimate the temperature of the experimental shocks. They deduce from this approach a melting point of $MgSiO_3$ at 500 GPa and 9430 K, i.e. at much higher temperature than expected based on lower pressure measurements.

A thermal equation of state is then fitted to the data in the solid domain and used to construct a new mass-radius relation for $MgSiO_3$ that is applicable to the mantle of super-Earth up to 8 Earth-masses. Using this relation, the authors aim at establishing a reference mantle density profile based on $MgSiO_3$. Its implications regarding the structure and composition of various exoplanets are discussed. Finally, the authors discuss the implications of the high-temperature melting curve of $MgSiO_3$ on the thermal evolution of the deep mantle and core of super-Earths and on the development and maintaining of a dynamo.

General comments:

This well-written manuscript presents new and very high-quality experimental data (as expected for this large facility) in a quasi-unexplored range of thermodynamic conditions relevant for super-Earth's interiors. Given the ever-increasing number of exoplanet detections, the needs for accurate equations of state of planetary interior components are growing, this study is timely and present a potential interest for several scientific communities (high-pressure science, planetology, geodynamics, …). I would like to add that all the details given in Methods and Supplementary Materials are very much appreciated.

Prior recommending publication, I would like to discuss several points with the authors, especially regarding certain underlying assumptions that should be more clearly stated in the text. The second main point concerns the determination of the melting line based on the sound velocity measurements.

Main comments:

1) Assumptions regarding the structural state of the shocked sample and thermodynamic equilibrium.

The experimental setup employed in this study does not give any direct information regarding the structural state of the sample. The comparison DFT-MD data is used to suggest that shocked Bd is transformed in a post-perovskite phase between 294 and 500 GPa. This motivates the use of the DFT-MD Ppv Hugoniot temperatures below melting.

To my opinion, it is not obvious that this argument can be made for the following reasons:

• Given the proximity of the computed Bd, Ppv and liquid/glass (I am pretty sure that the simulations of disordered $MgSiO_3$ at 2223 and 5505 K are in a glassy state) Hugoniots in the pressure-density space, the experimental data between 294 and 500 GPa could match both Bd (metastable), Ppv or

liquid/glass state (metastable).
• The longitudinal velocity of metastable Bd and Ppv are likely similar, and the longitudinal velocity of the glass is not known.
Therefore, by suggesting that shocked Bd transforms to Ppv, the authors implicitly say that the equilibrium phase is reached during the 20-40 nanoseconds of the shock. However, a recent DAC study has shown that it takes several hundred seconds to transform Bd into Ppv under static compression at 1850 K and 126 GPa (Langrand et al., Kinetics and detectability of the bridgmanite to post-perovskite transformation in the Earth's D” layer, Nature Communications 2019).
However, if bridgmanite amorphizes during the shock compression and forms a glass state, the temperature of the shock would be much lower and the transition around 500 GPa would be a glass-liquid transition. Such amorphization has recently been observed by ultrafast XRD in laser-shocked MgSiO3 orthoenstatite (Hernandez et al., Direct Observation of Shock-Induced Disordering of Enstatite Below the Melting Temperature, GRL 2020).

Based on the kinetics of Bd-Ppv transformation and assuming that in MgSiO3 glass the difference between the longitudinal and bulk velocities is smaller than what has been measured in the present experiment, I would say that the shocked state remains bridgmanite up to the melting. In this case, non-equilibrium conditions are probed in the solid, but given the proximity of the computed Bd and Ppv Hugoniots in a pressure-temperature space, the thermal EOS and mass-radius relations would be similar if considering either Bd or Ppv, and this would not have any effect on the conclusion drawn in the paper regarding planetary interiors.

I would like the authors to answer my comment and discuss the possible interpretations. I also suggest to clearly state in the main text the underlying assumptions regarding the structural state of shocked Bd and the notion of thermodynamic equilibrium that is important for applications to planetary interiors.

2) Melting curve of MgSiO3.

An important point of this paper is the high-temperature melting curve of MgSiO3. However, I do not understand why the author consider shot Z3029 as being purely solid (in Fig. 2) and not in the mixed phase region as it is written at the end of page 6: “With the shot Z3029 in the mixed phase region, we place…”.

This choice strongly influence the melting temperature and considering that in shot Z3029 Vp is smaller than in shot Z3116, I would have chosen shot Z3116 as the last pure solid. In this case, melting starts around 422 GPa and ends around 550 GPa, which is also more consistent with the pressure dependence of Vp decrease between shots Z3116 and Z3029. The resulting melting curve would be much more consistent with the recent measurement of Fratanduono et al., Thermodynamic properties of MgSiO3 at super-Earth mantle conditions, PRB 2018. The resulting discrepancy with Luo et al., Shock temperature and melting of silicates, Journal of Geophysical Research 2004, would not be an issue because Luo et al. measurements imply a decrease in density at the solid-liquid transition which is inconsistent with all other experimental and theoretical studies published since then.

3) MgSiO3 ppv is expected to dissociate into Mg2SiO4 + MgSi2O5 around 0.7 TPa according to first-principles simulations (Umemoto et al., Phase transitions in MgSiO3 post-perovskite in super-Earth mantles, EPSL 2017). How such dissociation would affect the mass-radius relation derived in this paper?

4) In Fig. 1, do you have any explanation for the discrepancy between the calculated Hugoniot of Ppv and data points from Mosenfelder et al. 2009? How Us-Up data in shocked bridgmanite from this study compare with the ones from Deng et al. PEPI 2008, Mosenfelder et al., JGR 2009 and Millot et al. GRL 2020?

5) Page 9: I find the discussion about the implications that a high-melting melting curve of $MgSiO_3$ could have on the generation of a dynamo a bit speculative. We do not know what are the chemical exchanges between the core and the mantle at pressures and temperatures of super-Earths' CMB and the development of a dynamo on Earth is also not very well understood. Even if I agree with the logic of the reasoning I would tone this part down.

Other comments:

• Introduction: "Typical dynamic compression experiments use $MgSiO_3$ enstatite and glass[2,3,19,20] as the starting materials, with initial densities < 3.2 g/cm3.".

Two recent studies should be referenced here: 1) Morard et al., In situ X-ray diffraction of silicate liquids and glasses under dynamic and static compression to megabar pressure, PNAS 2020; 2) Hernandez et al., Direct observation of shock-induced disordering of enstatite below the melting temperature, GRL 2020.

• Introduction: "Very recently, pre-synthesized $MgSiO_3$ bridgmanite has been shocked to hot liquid at 1150-1430 GPa by the laser-driven shock technique[18], but no direct detection of melting and density measurements of the solid phase along the bridgmanite Hugoniot."

This sentence is not complete.

• Please precise if bridgmanite samples are polycrystals or single crystals.

• End of page 4 and first line of page 5: "We analysed the sound velocity of these shots using multi-sample technique that leverages the different bridgmanite sample thicknesses produced different window overtake times."
Should it be "We analysed the sound velocity of these shots using multi-sample technique that leverages the different bridgmanite sample thicknesses AND produceS different window overtake times."?

• Fig. 2's caption, panel (a): "blue dotted line" should be "purple dotted line".

• Last line of page 6: "For the first time, we obtained high-quality experimental data under extreme conditions that are used as benchmarks for theoretical calculations."

Technically this is not the first time since Millot et al., GRL 2020 have probed liquid $MgSiO_3$ up to 1.4 TPa.

• Beginning of page 8: "Our calculated results represent… …, without any pressure extrapolation, because the core-mantle boundary pressure for a super-Earth with 8 ME is about 1200 GPa."

If I understood correctly, the thermal EOS is only for the solid state, but the measurements in the solid reach 400-500 GPa. So, the thermal EOS is actually extrapolated up to 1200 GPa in the solid state. This is an extrapolation of 700 GPa. In this case, the corresponding sentence should be corrected.

• The notion of potential mantle temperature appears several times through the manuscript and should be defined. I guess it is the temperature that would have the mantle adiabat if it was extrapolated to the surface.

• Experimental methods: "Adjacent samples were transparent quartz or diamond/quartz windows

which enabled observation of the flyer plate and determination of the impact time at the sample.".

If the impact time is not measured on the bridgmanite samples, I guess the Bd samples are not transparent and are polycrystals? Moreover, all targets located on the same side of the anode must have the same impact time. Is there any error associated with this alignment?

• Is there any other explanation for the initial release in shot Z3203 (and other shots) than an edge rarefaction?

• Why is the y-axis "Particle velocity" in Fig. S3 (c)?

Regarding first-principles simulations, at a given pressure the cell shape was optimized at 0 K and then molecular dynamics was run in the NVT ensemble at different temperatures using the optimized same cell shape. Therefore, the authors assume that the temperature does not influence the cell parameter ratios in Bd and Ppv. Is this assumption reasonable?

Responses to REVIEWER COMMENTS (our responses in blue), followed by showing all changes in the manuscript file with track changes

Reviewer #1 (Remarks to the Author):

Bridgmanite is the most abundant mineral in Earth's silicate mantle and is almost certainly an important constituent of rocky exoplanets. The melting curve of bridgmanite at extremely high temperatures is critical to models of planetary structure and evolution. However, experiments that directly measure melting temperatures at extreme conditions are difficult and scarce. This compelling, well-written manuscript reports new data from shockwave loading of pre-synthesized bridgmanite. The melting temperature at very high pressures is higher than expected based on prior studies, which could have important implications for the likelihood that accretional heating melts the mantle and the subsequent vigor of mantle convection.

I have one concern about the interpretation of the experimental data, which I think stems from my inexperience with actually performing shock experiments. Hopefully, addressing this concern will make the manuscript more digestible for a broader audience. I also have a few minor comments about the general framing of the study. Assuming the authors address these comments, I enthusiastically recommend publication.

Moderate comment:
As a non-practitioner of shock experiments, I was confused about some of the implications of using "dense MgSiO3" in the experiments. I suggest quoting the actual pressure and temperature conditions at which the samples were synthesized in the main text. Currently, "lower-mantle pressures" are mentioned in page 2, but it would be worth quoting 25 GPa and 1673 K (as noted in the Methods). Basically, I would clarify for the general reader that the experimental samples are ~33% denser than usual because they have already been subjected to inelastic compression, and not because of porosity or compositional effects.
As suggested, we clarify the nature of the starting material in terms of density and synthesis conditions.

*More importantly, I think the methods description on pages 16 and 17 is a bit incomplete. Specifically, what is the reference pressure (P0) used in Eq. 5 (Rankine-Hugoniot relation) and the following equations? Page 16 states that it "was obtained from the energy and pressure of a well-equilibrated DFTMD calculation of bridgmanite at 300 K with a density of 4.1 g/cc." However, I did not see the reference pressure explicitly quoted in the text, Table S1, Figure 1. I *assume* that reference pressure close to the synthesis pressure (25 GPa)? I presume that "dense MgSiO3" has a higher (?) melting temperature at ambient pressure than regular MgSiO3 with a density of ~3.2 g/cc. So, if ambient pressure was used as a reference pressure, then the melting temperatures and densities of real bridgmanite in planetary mantles would be over(?)estimated?*
We have accidentally omitted the reference state from Table S1. Thank you for pointing that out. It has been added. In these experiments the reference state for both the experiments and DFTMD is 4.1 g/cc and 300 K because that is the initial state before the shock wave propagates through the sample.

Minor comments:
- On page 2, the last part of the last sentence of the first full paragraph ("no direct detection of melting and density…") is incomplete, missing a verb at least.
Changed to "but no direct detection of melting was reported, nor was the density of the solid phase along the bridgmanite Hugoniot measured"

- On page 6, the last full paragraph states "Given the excellent agreement of the calculated Hugoniot densities with the measurements, we place high confidence of [sic] the calculated Hugoniot temperatures." Please quantify "excellent" and "high confidence."
It references the comparison between the density measurements and DFTMD simulations shown in Figure 1. Modified the sentence to "Given the excellent agreement of the calculated Hugoniot densities with the measurements (c.f. Fig. 1), the calculated Hugoniot temperatures should be representative to the true values although the actual temperatures were not directly measured by the experiments."

- On page 8, this discussion should probably note that planets with radii above ~1.5 Earth-radii generally have thick volatile envelopes. 55 Cancri e is a bit of a special case since may have suffered substantial atmospheric loss given its close, hot orbit.
Thanks for the suggestion. We added a discussion.

- On page 9, I don't think "homologous law" is a common term in Earth and planetary science. Please add a citation/elaboration.
Changed to "the mantles of massive planets could be more viscous, resulted from the high melting temperature, which would reduce the core cooling rate.".

- On page 9, I think you should relatedly emphasize that this study does not directly compute (correct?) the viscosity of MgSiO3 in super-Earths. The possible temperature-dependence is described, but there also could be pressure effects expressed as a non-zero activation volume (correct?). I think the speculation about mantle dynamics is reasonable, but there are still lots of uncertainties about viscosities within massive planets.
Thanks for the suggestion. We agree the there are lots of uncertainties about viscosities at high pressure and temperature. We emphasized that the qualitative discussion is meant to illustrate the possible consequence on dynamo generation due to change of mantle properties with a high melting temperature.

Reviewer #2 (Remarks to the Author):

The paper "Melting and density of MgSiO3 determined by shock compression of bridgmanite to 1254 GPa" is a deep and complex paper that goes beyond the "simple" shock compression experiment as suggested by the title. From sample preparation to shock experiments, DFTMD simulations and a long discussion about possible implications for our knowledge of the internal structure and dynamics of super-Earths, it is obvious that a lot of care has been into every single part of the work presented here. Despite being rich and complex, the paper is for the most part rather easy to read, except perhaps the discussion that is difficult to fully understand without a careful read of a previous paper by the same team ("Super-Earth Internal Structures and Initial Thermal States" by Boujibar et al. 2020). This paper definitely presents brand new results that

will be of major interest to a wide audience. My recommendation is to publish it after minor revisions.
Thanks for the positive impressions of the work.

The shock experiments give very interesting results and the data quality is very high. There are however two limitations that are not discussed:

- absence of structural measurements: the evolution of the structure of the bridgmanite to ppv and perhaps other phases (a point that is discussed in length in Boujibar et al. 2020 but not here, we'll see that later) cannot be tracked with this technique ("Our experiments are expected to have shocked the sample into the stability field of post-perovskite and then into liquid state along the Hugoniot." p. 4). Data fits the lowest P data and are in agreement with simulations that's right but nonetheless there is no direct evidence that the ppv phase is the stable phase up to 500 GPa and ~ 9000 K. Moreover, the ppv phase is known for its strong anisotropic elastic and rheological properties. What happens during a strong uniaxial shock event? For example, can we expect some preferential orientation of the ppv? As this can influence the measurements done by the authors it would have been interesting to see this issue discussed.
That is a good point. We recognize the limitation due to absence of structural measurements. Density measurements alone would not be able to make any inference because the density change across the transition is very small (~1%). We explicitly stated the ppv phase is assumed. We added additional discussion to make the point clear and discussed possible future experiment to resolve the issue by laser-driven shock with in-situ X-ray diffraction measurement which is currently not possible at the Z-facility. It is difficult to address preferential orientation of the ppv. We added some discussion on possible influence of orientation-dependent strength on melting.

- absence of T measurements. All T reported here are calculated. On p. 6 the authors' claim about the accuracy of the estimation appears however reasonable. P. 6 is pretty critical because it is mostly about the onset of melting recorded at 500 GPa, a very important measurement. That's a pity that T couldn't be measured with a streak camera. Looking at Figure S1 it seems pretty difficult to have an optical access to at least one sample, but one can hope for the future. A brief comment about why it couldn't be done would be interesting.
The Sandia diagnostic team is working toward temperature measurements, but the sample requirements are very specific. The synthetic bridgmanite sample is not sufficiently transparent to even do any test measurements.

The discussion about super-Earths internal structure, "mantle density excess", core solidification and deep mantle viscosity is an interesting read, though difficult to fully follow without carefully reading Boujibar et al. 2020. Three comments:

- H2O content of exoplanet 55-Cnce p. 8: I understand the exercise here but the uncertainties go far beyond the error bars of Fig. 3a. Perhaps a bit more careful phrasing should be used.
Thanks for the suggestion. We modified few sentences to just illustrate a possible alternative explanation based on the new density measurements. We did point out the caveat that "there are compositional degeneracy and large observational uncertainty to be considered for a detailed model".

- where does the mantle potential T value of 1725 K come from?
A large range in ambient mantle potential temperature values, from 1573 K to 1843 K, has been proposed on the basis of the petrology and geochemistry of erupted lavas. We added a reference by *Hertzberg et al.* (2007) who provided an extensive discussion.

- extrapolation of MgSiO3 melting curve beyond the probable stability field of MgSiO3 ppv. The phase transition from ppv to post-ppv phases is not discussed in this paper but it is in Boujibar et al. 2020. In the P range considered (up to 1.2 TPa) several scenarios are possible. Perhaps this has no effect on the shape of the melting curve, perhaps there are no liquid-liquid transitions to be expected. But I think this could be clarified by the authors.
We cited the results of *Boujibar et al.* (2020), and added a brief discussion.

Reviewer #3 (Remarks to the Author):

Summary:

In this manuscript, Fei et al. investigate the equation of state and melting of MgSiO3 between 296 and 1254 GPa. They report plate-impact shock-compression experiments on pre-synthetized bridgmanite samples carried out at Sandia National Laboratory Z machine.

The pressure and density along the bridgmanite Hugoniot are calculated from the measured mean shock velocity (Us) and flyer plate velocity (Uf) based on impedance matching procedures and Rankine-Hugoniot relations. Moreover, longitudinal and bulk sound velocity measurements are performed using the overtaking rarefaction approach. The absence of longitudinal sound velocity above 500 GPa leads the authors to conclude that melting occurred at 500 GPa along the Hugoniot.

These experimental results are compared to new first-principles atomistic simulations (DFT-MD) of bridgmanite, post-perovskite and liquid MgSiO3. Based on the good agreement between the experimental and numerical results (Hugoniot in the pressure-density space and bulk sound velocity), the authors used the DFT-MD Hugoniot temperatures to estimate the temperature of the experimental shocks. They deduce from this approach a melting point of MgSiO3 at 500 GPa and 9430 K, i.e. at much higher temperature than expected based on lower pressure measurements.

A thermal equation of state is then fitted to the data in the solid domain and used to construct a new mass-radius relation for MgSiO3 that is applicable to the mantle of super-Earth up to 8 Earth-masses. Using this relation, the authors aim at establishing a reference mantle density profile based on MgSiO3. Its implications regarding the structure and composition of various exoplanets are discussed. Finally, the authors discuss the implications of the high-temperature melting curve of MgSiO3 on the thermal evolution of the deep mantle and core of super-Earths and on the development and maintaining of a dynamo.
Thank you for the excellent summary and the constructive suggestions.

General comments:

This well-written manuscript presents new and very high-quality experimental data (as expected for this large facility) in a quasi-unexplored range of thermodynamic conditions relevant for super-Earth's interiors. Given the ever-increasing number of exoplanet detections, the needs for accurate equations of state of planetary interior components are growing, this study is timely and present a potential interest for several scientific communities (high-pressure science, planetology, geodynamics, ...). I would like to add that all the details given in Methods and Supplementary Materials are very much appreciated.

Prior recommending publication, I would like to discuss several points with the authors, especially regarding certain underlying assumptions that should be more clearly stated in the text. The second main point concerns the determination of the melting line based on the sound velocity measurements.

Main comments:

1) Assumptions regarding the structural state of the shocked sample and thermodynamic equilibrium.

The experimental setup employed in this study does not give any direct information regarding the structural state of the sample. The comparison DFT-MD data is used to suggest that shocked Bd is transformed in a post-perovskite phase between 294 and 500 GPa. This motivates the use of the DFT-MD Ppv Hugoniot temperatures below melting.

To my opinion, it is not obvious that this argument can be made for the following reasons:

- *Given the proximity of the computed Bd, Ppv and liquid/glass (I am pretty sure that the simulations of disordered MgSiO3 at 2223 and 5505 K are in a glassy state) Hugoniots in the pressure-density space, the experimental data between 294 and 500 GPa could match both Bd (metastable), Ppv or liquid/glass state (metastable).*
- *The longitudinal velocity of metastable Bd and Ppv are likely similar, and the longitudinal velocity of the glass is not known.*

Therefore, by suggesting that shocked Bd transforms to Ppv, the authors implicitly say that the equilibrium phase is reached during the 20-40 nanoseconds of the shock. However, a recent DAC study has shown that it takes several hundred seconds to transform Bd into Ppv under static compression at 1850 K and 126 GPa (Langrand et al., Kinetics and detectability of the bridgmanite to post-perovskite transformation in the Earth's D" layer, Nature Communications 2019).
However, if bridgmanite amorphizes during the shock compression and forms a glass state, the temperature of the shock would be much lower and the transition around 500 GPa would be a glass-liquid transition. Such amorphization has recently been observed by ultrafast XRD in laser-shocked MgSiO3 orthoenstatite (Hernandez et al., Direct Observation of Shock-Induced Disordering of Enstatite Below the Melting Temperature, GRL 2020).

Based on the kinetics of Bd-Ppv transformation and assuming that in MgSiO3 glass the difference between the longitudinal and bulk velocities is smaller than what has been measured in the present experiment, I would say that the shocked state remains bridgmanite up to the melting. In this case, non-equilibrium conditions are probed in the solid, but given the proximity of the computed Bd and Ppv Hugoniots in a pressure-temperature space, the thermal EOS and mass-radius relations would be similar if considering either Bd or Ppv, and this would not have any effect on the conclusion drawn in the paper regarding planetary interiors.

I would like the authors to answer my comment and discuss the possible interpretations. I also suggest to clearly state in the main text the underlying assumptions regarding the structural state of shocked Bd and the notion of thermodynamic equilibrium that is important for applications to planetary interiors.

We thank the review for raising an important question regarding the structural state of the shocked sample and thermodynamic equilibrium. In the absence of structural measurement, we do not have the ability to conclusively state if the sample transformed to post-perovskite. In the revision, we explicitly stated the Ppv phase is assumed. It is reasonable to make this assumption because our experiment at the lowest pressure (296 GPa) has reached the conditions significantly above the bridgmanite-ppv transition boundary along hot Hugoniot temperature and the agreement between the measurement and simulation is excellent. There is active pursue by several groups to determine the structural state of the shocked sample with in-situ X-ray diffraction measurements, and we are also actively proposing structural measurements at laser-shock facilities using bridgmanite starting material, hopefully to shed light on this issue in the near future. The result on shock-induced disordering by *Hernandez et al* is very interesting, but may not applicable to our experiments because we used denser bridgmanite as starting material which has the advantage of bypassing the low-pressure phase transitions. The timescale of the laser-driven shock and the magnetically driven plate-impact experiments is also very different, that could affect the outcome. It is premature to discuss alternative explanations in this study, although we are also very interesting in understanding the issue, particularly using bridgmanite as the starting material. At this stage, we prefer not to speculate if bridgmanite remains metastable up to melting or transforms to glass. As far as density models concerned, the structure state would not have any effect on the conclusions related to the mass-radius relations as pointed out by the reviewer.

2) Melting curve of MgSiO3.

An important point of this paper is the high-temperature melting curve of MgSiO3. However, I do not understand why the author consider shot Z3029 as being purely solid (in Fig. 2) and not in the mixed phase region as it is written at the end of page 6: "With the shot Z3029 in the mixed phase region, we place…".

This choice strongly influence the melting temperature and considering that in shot Z3029 Vp is smaller than in shot Z3116, I would have chosen shot Z3116 as the last pure solid. In this case, melting starts around 422 GPa and ends around 550 GPa, which is also more consistent with the pressure dependence of Vp decrease between shots Z3116 and Z3029. The resulting melting curve would be much more consistent with the recent measurement of Fratanduono et al.,

Thermodynamic properties of MgSiO3 at super-Earth mantle conditions, PRB 2018. The resulting discrepancy with Luo et al., Shock temperature and melting of silicates, Journal of Geophysical Research 2004, would not be an issue because Luo et al. measurements imply a decrease in density at the solid-liquid transition which is inconsistent with all other experimental and theoretical studies published since then.
This this a valid argument and it deserves further discussion. We added some discussions in the revision. We agree that shot Z3116 at 422 GPa is definitely at solid state with a calculated Hugoniot temperature of 7240 K, which is higher than the extrapolated melting temperature of Fratanduono et al. (c.f. Fig. 2b). In the absence of structural measurement, we do not have the ability to conclusively state whether the decrease in longitudinal sound velocity is from partial melting and entering a mixed phase or a softening of the shear modulus when approaching melt. It is conceivable that bridgmanite has orientation-dependent strength, which can manifest in melting occurring at different shock pressures. This could result in solid bridgmanite pockets within a liquid medium, where the individual solids could possibly support longitudinal and bulk waves. In the revision we stated that by placing shot Z3029 along the solid Hugoniot, the inferred melting temperature would represent an upper bound. We added further discussion on melting temperature by assuming a mixed phase. The inferred melting temperature would be about 1000 K lower than the upper bound and it brings to a better agreement with the theoretical calculation, but still higher than the extrapolation of the laser-shock melting from enstatite starting material. The conclusion of "high melting temperature" from this study is still valid. The melting temperature discussion has minimal impact on the discussion of early melting processes because we concluded that extensive mantle melting in super-Earths would be common following accretion even with our upper-bound melting curve.

3) MgSiO3 ppv is expected to dissociate into Mg2SiO4 + MgSi2O5 around 0.7 TPa according to first-principles simulations (Umemoto et al., Phase transitions in MgSiO3 post-perovskite in super-Earth mantles, EPSL 2017). How such dissociation would affect the mass-radius relation derived in this paper?
We have explored this question in our recent paper (Boujibar, et al., 2020) and demonstrated the effect on the mass-radius relation is miniscule.

4) In Fig. 1, do you have any explanation for the discrepancy between the calculated Hugoniot of Ppv and data points from Mosenfelder et al. 2009? How Us-Up data in shocked bridgmanite from this study compare with the ones from Deng et al. PEPI 2008, Mosenfelder et al., JGR 2009 and Millot et al. GRL 2020?
The discrepancy between the calculated Hugoniot of Ppv and data points from Mosenfelder et al. (2009) is due to the lower initial density of the Mosenfelder sample that contains some majorite component. The Us-Up data are in a general agreement. We added a plot to show the Us-Up relationship for bridgmanite starting material in the Supplementary Materials (Fig. S2).

5) Page 9: I find the discussion about the implications that a high-melting melting curve of MgSiO3 could have on the generation of a dynamo a bit speculative. We do not know what are the chemical exchanges between the core and the mantle at pressures and temperatures of super-Earths' CMB and the development of a dynamo on Earth is also not very well understood. Even if I agree with the logic of the reasoning I would tone this part down.

We agree this is a rather qualitive discussion. We emphasized the discussion is meant to illustrate the importance for understanding the mantle melting temperature and possible implications.

Other comments:

• *Introduction: "Typical dynamic compression experiments use MgSiO3 enstatite and glass2,3,19,20 as the starting materials, with initial densities < 3.2 g/cm3.".*

Two recent studies should be referenced here: 1) Morard et al., In situ X-ray diffraction of silicate liquids and glasses under dynamic and static compression to megabar pressure, PNAS 2020; 2) Hernandez et al., Direct observation of shock-induced disordering of enstatite below the melting temperature, GRL 2020.
We added the new references in the revision.

• *Introduction: "Very recently, pre-synthesized MgSiO3 bridgmanite has been shocked to hot liquid at 1150-1430 GPa by the laser-driven shock technique18, but no direct detection of melting and density measurements of the solid phase along the bridgmanite Hugoniot."*

This sentence is not complete.
Changed to "no direct detection of melting was reported, nor was the density of the solid phase along the bridgmanite Hugoniot measured"

• *Please precise if bridgmanite samples are polycrystals or single crystals.*
We added that the bridgmanite samples are polycrystals.

• *End of page 4 and first line of page 5: "We analysed the sound velocity of these shots using multi-sample technique that leverages the different bridgmanite sample thicknesses produced different window overtake times."*
Should it be "We analysed the sound velocity of these shots using multi-sample technique that leverages the different bridgmanite sample thicknesses AND produceS different window overtake times."?
Change the sentence to "We analyzed the sound velocity of these shots using multi-sample technique which leverages that the different bridgmanite sample thicknesses produced different window overtake times."

• *Fig. 2's caption, panel (a): "blue dotted line" should be "purple dotted line".*
Changed.

• *Last line of page 6: "For the first time, we obtained high-quality experimental data under extreme conditions that are used as benchmarks for theoretical calculations."*

Technically this is not the first time since Millot et al., GRL 2020 have probed liquid MgSiO3 up to 1.4 TPa.
Here we try to emphasize the EOS of solid for modeling the solid mantle density profile. We modified the sentence to "*For the first time, we obtained high-quality density measurements of*

solid $MgSiO_3$ under extreme conditions that are used to as benchmarks for theoretical calculations."

• *Beginning of page 8: "Our calculated results represent… …, without any pressure extrapolation, because the core-mantle boundary pressure for a super-Earth with 8 ME is about 1200 GPa."*

If I understood correctly, the thermal EOS is only for the solid state, but the measurements in the solid reach 400-500 GPa. So, the thermal EOS is actually extrapolated up to 1200 GPa in the solid state. This is an extrapolation of 700 GPa. In this case, the corresponding sentence should be corrected.
We reworded the sentence to reflect the nature of the extrapolation. The new sentence reads as "*Our calculated results represent the best estimated density profile for a model $MgSiO_3$ mantle of super-Earths up to 8 ME, with combination of direct density measurements and ground-truthed DFTMD calculations*".

• *The notion of potential mantle temperature appears several times through the manuscript and should be defined. I guess it is the temperature that would have the mantle adiabat if it was extrapolated to the surface.*
Correctly stated. It is the temperature if adiabatically brought to the surface from mantle depth without undergoing melting. We added a reference by *Hertzberg et al*. (2007) who provided an extensive discussion.

• *Experimental methods: "Adjacent samples were transparent quartz or diamond/quartz windows which enabled observation of the flyer plate and determination of the impact time at the sample.".*

If the impact time is not measured on the bridgmanite samples, I guess the Bd samples are not transparent and are polycrystals? Moreover, all targets located on the same side of the anode must have the same impact time. Is there any error associated with this alignment?
The Bd samples are not transparent. Impact time on the samples is determined by interpolation using the observed impact times onto transparent samples/windows above and below the Bd samples. Corrections are applied for dynamic flyer tilt (interpolation) and measured flight gap differences from sample to sample due to imperfect assembly/mounting of the target panel. The measured impact time difference from the top to the bottom of the panel is typically ~1-3 ns. Part of this is due to dynamic flyer bowing/tilt during acceleration and part due to variations in the flight gap distance between the initial flyer position and the samples (assembly and alignment precision). The flight gap correction is typically on the order of ~0.5 ns (resulting from ~10 micron differences in flight gap). These corrections are small, but important, and were applied for every Bd Hugoniot measurement presented using the unique above/below measured impact times and flight gaps.

• *Is there any other explanation for the initial release in shot Z3203 (and other shots) than an edge rarefaction?*
On shots 3203 and 3029 there is no viable explanation other than an edge wave for the first stage of a three-stage release. If it were a longitudinal wave, the velocity would be unrealistically large

and the flyer characteristics would not result in an initial wave prior to the release from the Cu/Al interface because the Cu coating is dense (>99% full density). We added a justification for the edge wave assignment.

• *Why is the y-axis "Particle velocity" in Fig. S3 (c)?*
For consistency, we re-plotted Figure S3c using shock velocity as the y-axis.

Regarding first-principles simulations, at a given pressure the cell shape was optimized at 0 K and then molecular dynamics was run in the NVT ensemble at different temperatures using the optimized same cell shape. Therefore, the authors assume that the temperature does not influence the cell parameter ratios in Bd and Ppv. Is this assumption reasonable?
The reviewer is correct that we enforce constant lattice parameter ratios in the solid. To the best of our knowledge no experiments or theoretical calculations to date have quantified the degree of anisotropic thermal expansion in Ppv, or for any silicate mineral at high pressure for that matter. We also did not investigate this. In the solid the thermal expansion is a tensor which is constrained to have the same symmetry as the lattice. And a general feature of the (averaged) thermal expansion of silicates is that it decreases as pressure increases. So, at the conditions of interest in the present work, the anisotropic thermal expansion should be very small. To make the assumptions in our calculation clear to the reader, we added "*For bridgmanite and post-perovskite, the lattice parameter ratios for the high-temperature DFTMD calculations were unchanged from their optimized 0 K values.*"

Melting and density of $MgSiO_3$ determined by shock compression of bridgmanite to 1254 GPa

Yingwei Fei[1,*], Christopher T. Seagle[2], Joshua P. Townsend[2], Chad A. McCoy[2], Asmaa Boujibar[1], Peter Driscoll[1], Luke Shulenburger[2], Michael D. Furnish[2]

[1]Earth and Planets Laboratory, Carnegie Institution for Science, 5251 Broad Branch Road, NW, Washington, DC 20015, USA
[2]Sandia National Laboratories, Albuquerque, NM 87185, USA
[*]Correspondence to: Yingwei Fei (yfei@carnegiescience.edu)

ABSTRACT

Formatted: Font: Bold

The essential data for interior and thermal evolution models of the Earth and super-Earths are the density and melting of mantle silicate under extreme pressure-temperature conditions. The melting of $MgSiO_3$-silicate, an important endmember for all rocky planet mantles, has been measured up to 62 and 183 GPa by static and dynamic compression methods, respectivly[1-3], and the density of the crystalline phase determined up to 260 GPa at room temperature and to 170 GPa at high temperature by X-ray diffraction in the diamond-anvil cell[4-7]. Measurements beyond these conditions are unattainable by conventional methods, thereby hampering our ability to make fundamental advances in the understanding of the interior structure and dynamics of super-Earths. Here, we report unprecedently high melting temperature of $MgSiO_3$ at 500 GPa by direct shockwave loading of pre-synthesized dense $MgSiO_3$ (bridgmanite) using

the Z Pulsed Power Facility. We also present the first high-precision density data of crystalline $MgSiO_3$ phase to at least 422 GPa and 7200 K and of silicate melt up to 1254 GPa. The experimental density measurements support our density functional theory based molecular dynamics (DFTMD) calculations, providing benchmarks for theoretical calculations under extreme conditions. The excellent agreement between experiment and theory provides a reliable reference density profile for super-Earth mantles. Furthermore, the observed upper bound of melting temperature, 9430 K at 500 GPa, provides a critical constraint on the accretion energy required to melt the mantle and the prospect of driving a dynamo in massive rocky planets.

INTRODUCTION

The Earth's lower mantle (extending to a pressure of 136 GPa) is primarily composed of bridgmanite[8], a dense high-pressure polymorph of $MgSiO_3$. $MgSiO_3$-bridgmanite further transforms to post-perovskite at the bottom of the lower mantle[9]. Accurate measurements of the density of bridgmanite and post-perovskite as a function of pressure, temperature, and composition, have been the major goal of the Solid-Earth research, using the static and dynamic compression methods[4-7,10-13], in order to decipher the composition of the mantle. The observed melting in the laser-heated diamond-anvil cell[1] and by shockwave methods[2,3] is still debated, because of the extreme temperature required to melt $MgSiO_3$-bridgmanite above 60 GPa. The mantle of a rocky exoplanet is likely composed of Mg-Fe silicate based on the observed stellar Fe, Mg and Si abundances[14,15]. These observations suggest the Mg/Si ratios between 0.67 and 1.5 centered at about 1.0, a $MgSiO_3$ composition. Therefore, $MgSiO_3$ is an important endmember for all rocky planet mantles. The pressure of the super-Earth mantle could exceed 1400 GPa when its mass reaches 10 Earth masses (M_E)[16]. Directly probing materials under such extreme conditions is now attainable only through dynamic compression techniques[17,18]. However, the access to the solid silicate phase along Hugoniot (a shock compression pressure-temperature path) and its melting at higher pressure strongly depends on the initial density of the starting materials.

Typical dynamic compression experiments use $MgSiO_3$ enstatite and glass[2,3,19-22] as the starting materials, with initial densities < 3.2 g/cm^3. These experiments suffer from the complication of phase transitions associated with large density changes (~28%) during shock compression and limited access to the solid phase region because of fast rising of Hugoniot temperature with shock pressure. The Hugoniot reaches melting temperature of $MgSiO_3$ below 180 GPa with the low-density starting materials[2,3]. Direct shock compression of pre-synthesized dense $MgSiO_3$ has been attempted up to 245 GPa by the traditional two-stage gas-gun technique[12,13]. Very recently, pre-synthesized $MgSiO_3$ bridgmanite has been shocked to hot liquid at 1150-1430 GPa by the laser-driven shock technique[18], but no direct detection of melting was reported, nor was the density of the solid phase along the bridgmanite Hugoniot measured.

In order to obtain density measurements of solid $MgSiO_3$ above 245 GPa and detect its melting at much higher pressure, in this study, we synthesized large polycrystalline bridgmanite sample in a multi-anvil device at 25 GPa and 1673 K, which is the densest recoverable $MgSiO_3$ mineral, with a density of 4.1 g/cm^3 under ambient conditions. The synthesis utilized the newly developed techniques that optimized sample volume at the synthesis condition using extremely large, strong sintered diamond cubes (25.4-mm) (see Methods). Using the synthetic $MgSiO_3$

Deleted: of
Deleted: ±600
Deleted: upper bound
Formatted: Font: Bold
Formatted: Indent: First line: 0.25"
Deleted: ,
Deleted: 20
Deleted: and
Deleted: measurements
Deleted: were reported
Deleted: by newly developed techniques that optimized sample volume at lower-mantle pressures using extremely large, strong sintered diamond cubes (25.4-mm). We used
Deleted: ,

bridgmanite as the starting material, we performed magnetically-driven plate impact experiments at the Sandia National Laboratories' Z Pulsed Power Facility. The bridgmanite Hugoniot allows us to measure the density and melting point of the solid phase to much higher pressure than any previous attempts. These new measurements are essential for developing reliable internal density structure of the solid super-Earth mantles and their thermal evolution.

Deleted: for the

RESUTLS

Formatted: Font: Bold

Hugoniot denisty measurements

Magnetically-accelerated flyers launched using the Z Pulsed Power Facility are capable of generating shock pressures over 1000 GPa[23,24]. The plate-impact experiments on the Z machine utilize the impedance-matching technique that allows high-precision determination of the Hugoniot states of the sample through the shock response of a known standard[23]. We carried out a total of 7 plate-impact experiments using the coaxial load geometry, with impact velocities ranging from 9.51 to 30.00 km/s. Pre-synthesized dense $MgSiO_3$ bridgmanite samples were used as the starting materials, whose structure of bridgmanite was confirmed by Raman spectra and X-ray diffraction, with a measured unit cell volume of 24.465±0.008 cm^3/mol (corresponding to a density of 4.103 g/cm^3). The samples were double-polished parallel to thicknesses from 0.3659 to 0.9339 mm, mounted in 5-mm Al-ring holder backed by quartz windows, 5-mm square with 1.5-mm thickness.

Deleted: GPa[21]

Deleted: [22]

Deleted: standard[21]

Supplementary Figure 1 shows the experimental configuration that consists of the north and south sample panels as impacting targets. The impacting target consists of 5-7 square sample holders with an edge length of 5 mm on each north or south panel. A sample diameter of at least 2 mm is required to ensure high-quality data. Each panel contains at least two bridgmanite samples while the remaining sample slots were used for the standards and other samples selected by the Z fundamental science program. Table 1 lists the details of the 7 bridgmanite experiments. Measurements of the four experiments (Z3011, Z3029, Z3116, and Z3203) were made using a symmetric coaxial target with a dual layer aluminum/copper flyer which resulted in identical impact velocity on the north and south sample panels. Each experiment provides 4 independent measurements under the same Hugoniot state, producing high-precision data. The experiments (Z3250 and Z3321) at the highest pressures used the same configuration but with an asymmetric A-K gap (Supplementary Fig. 1), which resulted in distinct impacting velocities on the north and south panels. The data precision for Z3250 is still high because three repetition measurements on bridgmanite were made at each panel. One sample on the north panel of Z3321 shot was compromised, resulting in an uncertainty of 2.6% in density measurements, instead of a typical uncertainty of ~1%. The experiment (Z3268) at the lowest pressure (296 GPa) of this study utilized an aluminum (Al) flyer impacting the two bridgmanite samples on the north panel only, producing comparable data quality (Table 1).

Deleted: S

Deleted: S

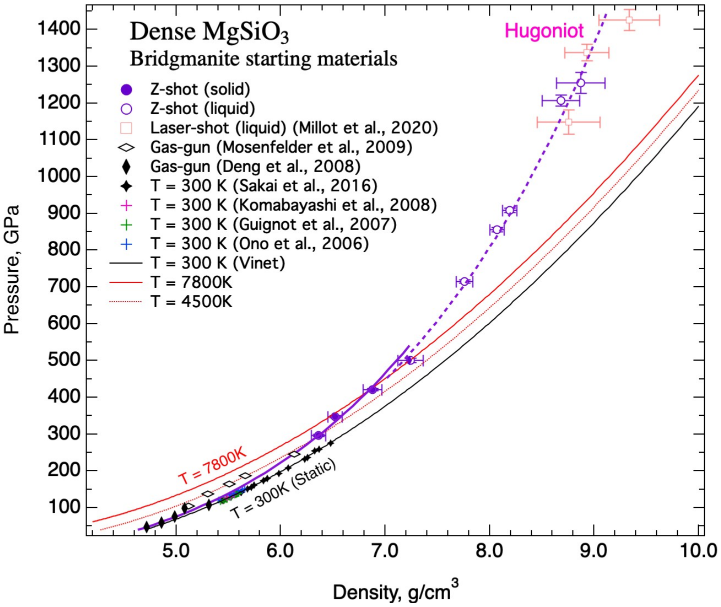

Figure 1. **Hugoniot data obtained using dense $MgSiO_3$-bridgmanite as the starting material.** The pressure-density data from magnetically-driven shock wave experiments in this study are shown with the purple circles. The orange open squares represent liquid $MgSiO_3$ Hugoniot data by the laser-driven shock[18]. The black solid and open diamonds represent compression data by gas-gun method[12,13]. The static compression data for $MgSiO_3$ post-perovskite at 300 K[4-7] are also shown for comparison. The purple solid and dashed curves are the calculated Hugoniots for post-perovskite and liquid, respectively, by DFTMD simulations. The calculated isotherms at 300, 4500 and 7800 K are shown with solid black, dashed red, and solid red curves, respectively.

Deleted: . 1
Formatted: Font: Not Bold
Formatted: Font: Bold

Velocity histories were measured by a velocity interferometer system for any reflector (VISAR)[25]. We used 38 channels of VISAR on each plate-impacting experiment, with fringe sensitivities ranging from 277 to 2293 m/s/fringe. Typically, data were recorded in 3 channels for each bridgmanite sample. The VISAR probe accurately tracks the velocity of the flyer from rest to impact, and records the shock history in bridgmanite sample and the quartz window. The bridgmanite shock velocity (U_s) was determined from the transit time for the shock to traverse the sample. The uncertainties in shock velocity are less than 2% based on the uncertainty in the transit time <0.7 ns. The particle velocity (u_p) and associated uncertainty were determined through impedance matching with the copper and aluminum flyer plate in a Monte Carlo technique which varied the equation of state of the flyer by ±3% and the flyer velocity, bridgmanite shock velocity, and bridgmanite initial density within 1%. Table 1 lists the measured shock and particle velocities, also shown in Supplementary Fig. 2. A least-squares fit to the data yielded a linear $U_s - u_p$ relation, $U_s = 7.922(\pm0.139) + 1.249(\pm0.015)u_p$.

Deleted: 23
Deleted: and the corresponding Hugoniot state of the bridgmanite with the initial density of 4.10 g/cm^3.

Figure 1 shows the density of $MgSiO_3$ as a function of pressure up to 1254 GPa along the Hugoniot. The Hugoniot data of the bridgmanite with the initial density of 4.10 g/cm^3 are listed in Table 1. Over the experimental pressure range the density increases more than twice from the initial bridgmanite. Under static compression, $MgSiO_3$-bridgmanite transforms to post-perovskite at ~125 GPa, with only about 1% density change[9]. Our experiments are expected to have shocked the sample into the stability field of post-perovskite and then into liquid state along the Hugoniot. We assumed the measured densities of the solid phase reflect those of post-perovskite because our experiment at the lowest pressure (296 GPa) has reached the conditions significantly above the bridgmanite-ppv transition boundary along hot Hugoniot temperature. In order to complement experimental data, we performed a series of *ab-initio* density functional theory (DFT) calculations for the bridgmanite, post perovskite, and liquid phases of $MgSiO_3$ (see Methods). Results are summarized in Supplementary Table 1. Our DFT based molecular dynamics (DFTMD) calculations follow a similar approach as in previous work[17]. The calculated density-pressure relation for post-perovskite along Hugoniot is shown by the solid purple curve which is in excellent agreement with the three low-pressure shots at 296, 348, and 422 GPa (Fig. 1), where the samples remained solid (see sound velocity data below). The calculated liquid Hugoniot pressure - density relation is shown by the dashed purple curve which is again in excellent agreement with the Hugoniot data between 500 and 1225 GPa. There is no liquid-liquid transition that can be detected from the liquid density data. The recent liquid Hugoniot data at 1150-1430 GPa by the laser-driven shock technique[18] are also shown for comparison. Within the uncertainties of the data, the laser-shock data are also plotted on the calculated curve. At the low-pressure end (<245 GPa), the gas-gun data[12,13], which were produced at the limit of the sample size, are also within the general trend.

Sound velocity and melting temperature determination

The DFTMD calculations define the Hugoniot temperature and pressure relations for the post-perovskite and the liquid phases (Supplementary Table 1). By determining the physical states (solid or liquid) of the shocked bridgmanite, we constrain the melting temperature of $MgSiO_3$ at extreme pressure. The standard way to detect melting in a dynamic compression experiment is via the sound velocities and the analysis of the release wave profiles. We determined the sound velocities using the overtaking rarefaction method with quartz windows[24,26]. In the overtaking rarefaction method, the back-propagating wave in the Cu impactor reflects from the Cu/Al interface as a release wave, which then propagates forward through the Cu impactor, bridgmanite sample, and quartz window until it overtakes the initial shock wave (Supplementary Fig. 3) (see Methods). The use of quartz windows decreases the uncertainty in the overtake time and enables additional analysis of the wave profile. Table 1 summarizes the results from the sound velocity analyses. Supplementary Figure 4 shows representative wave profiles, including a single-stage bulk release (plastic unloading) in liquid, a profile containing both longitudinal and bulk releases in the solid phase, and a complex profile showing edge wave in addition to the longitudinal and bulk releases in solid. The experiments (Z3011 at 715 GPa, Z3250S at 856 GPa, and Z3250N at 909 GPa) show simple single-stage bulk release. We analyzed the sound velocity of these shots using multi-sample technique which leverages that the different bridgmanite sample thicknesses produced different window overtake times. The measured sound velocities are consistent with the calculated bulk sound velocity of liquid (Fig. 2a). The calculated Hugoniot temperatures of these experiments are above

Deleted: Materials and

Deleted: S

Formatted: Font: Bold

Deleted: S

Deleted: windows[22]

Deleted: [24]

Deleted: S2

Deleted: S3

Deleted: that

11800 K (Supplementary Table 1) and they are expected to be shocked into the liquid state (Fig. 2b).

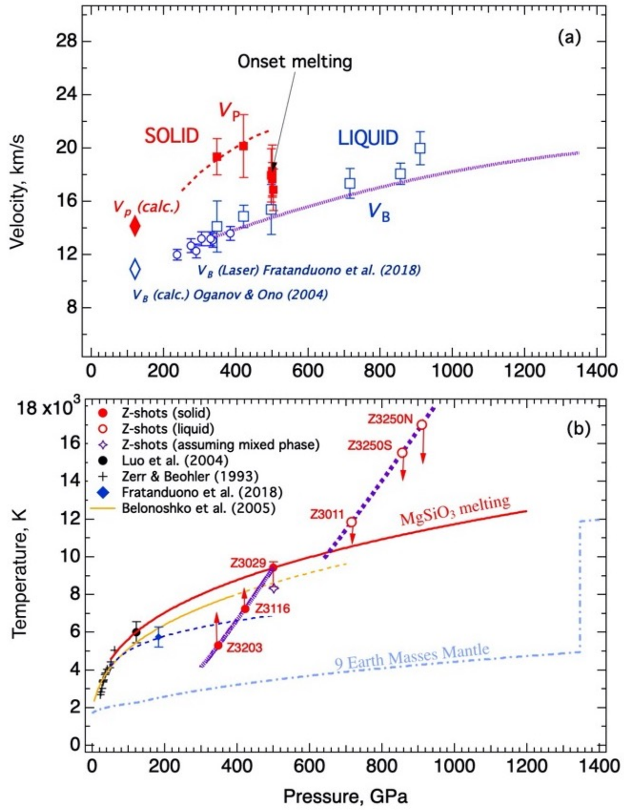

Figure 2. **Sound velocities (a) and melting temperature (b) of $MgSiO_3$ as a function of pressure. (a)** The bulk sound velocities (V_B) along Hugoniot are shown by the blue open squares, compared with theoretical calculations of this study (purple dotted line). The measured bulk sound velocities of liquid $MgSiO_3$ enstatite target by laser-driven shock[3] are also plotted for comparison. The longitudinal velocities (V_p) of the solid phase are shown by the red solid squares. The calculated values of V_p and V_B for post-perovskite at 125 GPa[27] are also shown for comparison. **(b)** The Z shots supporting longitudinal wave (red solid circles) are plotted on the calculated Hugoniot for solid post-perovskite (thick purple dotted line) whereas the experiments shocked into liquid are plotted on the calculated liquid Hugoniot (thick purple dashed line). The red curve represents the melting curve fitted to the upper bound melting constraint at 500 GPa along with low-pressure constraints from gas-gun compression of glass (black solid circle)[2] and static experiment (crosses)[1]. The vertical red arrows point toward melting temperatures. The open purple star indicates melting temperature assuming the shock entering a mixed phase. Theoretically calculated melting curve (yellow line)[28] and estimated melting point (blue diamond) and curve (blue dashed line) from laser-shock

Deleted: S

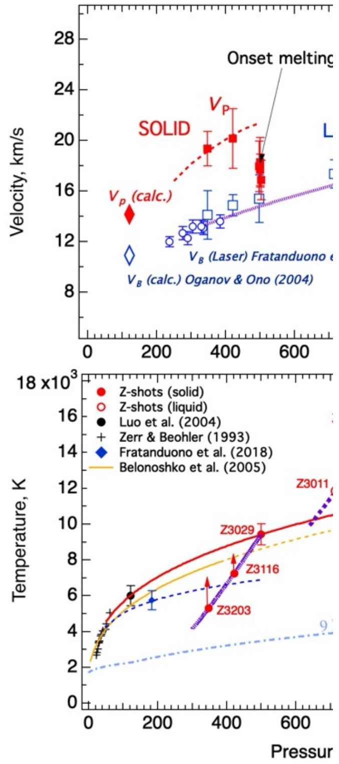

Deleted:

Deleted: .

Formatted: Font: Not Bold

Formatted: Font: Bold

Formatted: Font: Bold

Deleted: blue

Deleted: GPa[25]

Formatted: Font: Bold

Deleted: with

Deleted: the

Deleted: [26]

of enstatite[3] are shown for comparison. The temperature profile of super-Earth with 9 M_E used for density profile calculation is also shown.

The two experiments (Z3203 at 348 GPa and Z3116 at 422 GPa) show both longitudinal and bulk release waves. The measured bulk sound velocities agree well with the calculated bulk sound velocities along Hugoniot (Fig. 2a). We determined the longitudinal sound velocities of these two experiments from the longitudinal release profile using single-sample technique[24]. The values of the longitudinal sound velocity are about 36% higher than the bulk sound velocities because of the shear component of solid. Our data extrapolated to low pressure compare favorably to previous first-principles calculations[27].

The results from experiment Z3029 at 500 GPa are particularly interesting. All wave profiles show longitudinal release (Supplementary Fig. 5). Analysis of one bulk release indicates that the bulk sound velocity of this shot is consistent with the predicted value. However, the derived longitudinal velocities are substantially lower than the expected value for solid, but larger than the bulk sound velocity (Fig. 2a). The decrease in sound velocity is caused by shear softening likely related to melting, indicating an onset of melting.

For several wave profiles, the initial release can be attributed to an edge rarefaction impinging on the shock front in the center of the target prior to overtake of the longitudinal wave (Figs. S4c and S5). We find no viable explanation other than an edge wave for the first stage of a three-stage release, because if it were a longitudinal wave, the velocity would be unrealistically large and the flyer characteristics would not result in an initial wave prior to the release from the Cu/Al interface. The edge wave effect typically causes systematic overestimate of the sound velocity (see Methods) by about 3.5 %. This effect could explain the systematic offset between the observed and calculated sound velocities (Fig. 2a). Our calculated bulk sound velocities are also in good agreement with the measured bulk sound velocities of liquid $MgSiO_3$ at lower pressures by laser-shock technique, using enstatite as the target[3].

The temperatures of the shockwave experiments were not experimentally measured because the samples were not sufficiently transparent. We rely on DFTMD calculations (see Methods) to estimate Hugoniot temperatures at the shock pressures. The calculated Hugoniot temperatures at 500 GPa (shot Z3029) are 9430 K for post-perovskite and 6520 K for the liquid phase. The shocked bridgmanite is definitively at solid state at 422 GPa (shot Z3116), corresponding to a Hugoniot temperature of 7240 K, which is higher than the extrapolated melting temperature from laser-shock of enstatite[3], but lower than the calculated melting curve[28]. The liquid state is achieved at 715 GPa (shot Z3011), corresponding to a Hugoniot temperature of 11800 K. The wave profiles of the 500-GPa shot (Supplementary Fig. 5) show a clear longitudinal wave, indicating a solid phase component. By placing the shot along the solid Hugoniot, we obtain a melting temperature of $MgSiO_3$ at about 9430±600 K (Fig. 2). The inferred melting temperature would represent an upper bound. From the deduced sound velocity data, there is a decrease in longitudinal sound velocity (Fig. 2a). In the absence of structural measurement, we do not have the ability to conclusively state whether the decrease in longitudinal sound velocity is from partial melting and entering a mixed phase or a softening of the shear modulus when approaching melt. It is conceivable that bridgmanite has orientation-dependent strength, which can manifest in melting occurring at different shock pressures. This could result in solid bridgmanite pockets within a liquid medium, where the individual solids could possibly support longitudinal and bulk

Deleted: technique[22]

Deleted: calculations[25]

Deleted: S4

Deleted: S3

Deleted: S4

Deleted: s

Deleted: whereas t

Formatted: Superscript

Deleted: Based on the sound velocity measurements and the analysis of the wave profiles

Deleted: place the

Deleted: and 500 GPa

waves (Supplementary Fig. 5). Assuming a mixed phase, the inferred melting temperature would be about 8350 K by interpolating between the solid and liquid Hugoniot temperatures using a Poisson ratio of 0.37 based on the measured V_p and V_B values at 500 GPa (Fig. 2b). This temperature is about 1000 K lower than the upper bound and it brings to a better agreement with the theoretical calculation[28]. The measurement represents a direct detection of melting of silicate at such an extreme pressure and it is only possible because the dense silicate starting material allows us to access a cooler Hugoniot *P*-*T* path. Previous shock experiments with low-density starting materials reaches melting below 180 GPa[2,3].

Constrained by the melting temperatures at low pressure from static laser-heating experiments[1] and gas-gun shock compression[2], the upper bound of the $MgSiO_3$ melting curve can be expressed by the Simon power law, $T_m = 6295(P/140)^{0.317}$. This melting curve is substantially higher than the previously predicted melting curve up to 380 GPa by *ab initio* two-phase molecular dynamics simulations[28] and the estimated melting from laser-shock of enstatite[3] (Fig. 2b). The main uncertainty in the constrained melting temperature at 500 GPa comes from where to place the data along the Hugoniots. The inferred temperature could be 1000 K lower than the upper bound at 500 GPa as discussed above, resulting a lower melting curve, $T_m = 6000(P/140)^{0.26}$. This is still the highest experimentally determining melting. Given the excellent agreement of the calculated Hugoniot densities with the measurements (c.f. Fig. 1), the calculated Hugoniot temperatures should be representative to the true values although the actual temperatures were not directly measured by the experiments. The estimated melting temperature uncertainty from the DFTMD results is ±300 K given a melting pressure uncertainty of ±7 GPa.

Implications for super-Earth mantles

Accurate determination of the Hugoniot densities for $MgSiO_3$ from both experiments and theory provides a strong basis for establishing a reliable thermal equation of state that is applicable to density profile of the silicate mantle of super-Earths up to at least 8 *Me*. For the first time, we obtained high-quality density measurements of solid $MgSiO_3$ under extreme conditions that are used as benchmarks for theoretical calculations. By combining the static room-temperature compression data[4-7] with Hugoniot measurements and the calculated temperatures, we describe the pressure-density-temperature relations of $MgSiO_3$ solid by the Vinet equation of state[29] at 300 K with the thermal pressures calculated from the Mie-Grüneisen relation (see Methods), using the optimized parameters: ρ_0 = 4.176 g/cm^3, K_0 = 265.5 GPa, K_0' = 4.16, γ_0 = 1.57, q = 0.5, and θ_D=1000 K. With a model $MgSiO_3$ solid mantle of super-Earth and a mantle potential temperature of 1725 K, the calculated relation between mass and radius ratios is shown in Figure 3. The result of an iron core based on recent density measurements of pure iron up to 1400 GPa by laser-driven ramp compression[30] is also shown. The mass-radius relation for the Earth core/mantle fraction (CMF) is interpolated. The calculation did not include the effect of possible phase transformations beyond post-perovskite because these transformations have miniscule effect on the mass-radius relation as shown by our recently work[32].

Deleted: This is

Deleted: simulations[26]

Deleted: the calculation of Hugoniot temperature, since we have strong evidence for the shot at 500 GPa being at the solid-liquid boundary.

Deleted: we place high confidence of

Deleted: With the shot Z3029 in the mixed phase region, we place a general ~6% uncertainty in the melting temperature.

Deleted: experimental data

Deleted: state[27]

Deleted: compression[28]

Formatted: Superscript

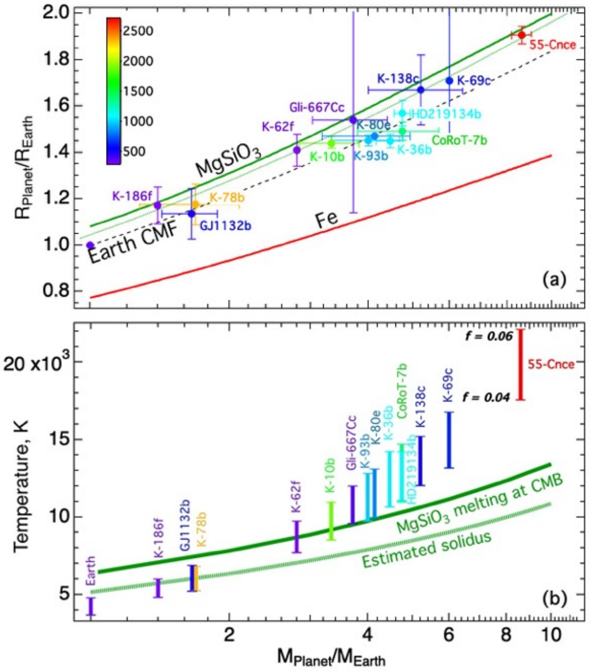

Fig. 3. Calculated radius ratios (a) and temperatures at the core-mantle boundary after accretion (b) as a function of mass ratios. (a) $MgSiO_3$ mantle is represented by green solid curve, pure Fe core by red solid curve[30], and $MgSiO_3$ – Fe planet with Earth CMF by black dashed curve. The calculated curve for an FeO-free Earth-like mantle[31] with Mg/Si = 1.07 (green dotted curve) is shown for comparison. Observations for selected super-Earths (www.exoplanet.eu) are shown with color scale indicating the estimated planetary equilibrium temperatures in Kelvin. Data without horizontal errors indicate that the mass is not well constrained. (b) Colored symbols represent the calculated CMB temperatures of the selected super-Earths, compared with $MgSiO_3$ melting curve and its estimated solidus temperatures calculated by $T_{solidus} = T_{melting}/(1 - \ln 0.79)$[33]. The lower bounds were calculated using the Earth-like $f = 0.04$ whereas the upper bounds using $f = 0.06$.

Our calculated results represent the best estimated density profile for a model $MgSiO_3$ mantle of super-Earths up to 8 M_E, with combination of direct density measurements and ground-truthed DFTMD calculations, because the core-mantle boundary pressure for a super-Earth with 8 M_E is about 1200 GPa. It is important to establish a reference mantle density profile using $MgSiO_3$ as an end-member component, as the mantle composition variations can be discussed using a concept of "mantle density excess" defined by the difference between the mantle density and the $MgSiO_3$ reference profile, similar to the "core density deficit" for the Earth's core. For the observed solar Fe, Mg and Si abundances[14,15], differentiated rocky planets would leave a silicate mantle enriched in iron although the amounts of Fe enrichment depend on the bulk iron content and the oxidation state during accretion. The mantle density excess

Deleted: curve[28]

Deleted: mantle[29]

Deleted: [30]

Deleted: out any pressure extrapolation

comparing to a $MgSiO_3$ reference would be a quantitative measure of the Fe-enrichment. Similarly, a silicate mantle with Mg/Si > 1 would lead to a denser mantle due to highly compressible MgO and transition of MgO to a denser CsCl structure in the deep mantle[34]. Strongly preferred Fe partitioning into the oxide would further increase the density. However, Silica-saturated mantle could have the opposite effect on density because high-pressure SiO_2 phases are relatively incompressible[35], even with the formation of the denser high-pressure pyrite-type phase[36].

The well-defined reference density model is a useful tool to track the chemical variations within the mantle. The mass-radius ratio relation of the $MgSiO_3$ mantle is also critical for understanding the core-mantle fraction and the presence or proportion of the H_2O or gas component in super-Earths. Our calculated mass-radius curve (Fig. 3) shifts up comparing to the commonly cited curve for $MgSiO_3$ [31] which significantly overestimated mantle density based on large extrapolation and theoretical calculations without benchmarks of experimental data. The difference could lead to different interpretation of the observations. For example, exoplanet *55 Cnc e* with a radius ratio of ~1.9, has a high planetary equilibrium temperature (~2709 K), and it plots at the different sides of the model mantle curve (Fig. 3). Planets with radius ratios above ~1.5 generally have thick volatile envelopes, but *55 Cancri e* may have suffered substantial atmospheric loss given its close, hot orbit. Being above the curve (dotted green) implies that the planet has to include light component such as H_2O which has to reconcile with the high planetary temperature according to previous model. Our result indicates that the observation could be simply interpreted by core-mantle fraction because it plots below our calculated curve (solid green). With the ideal end-member mantle and core compositions, the estimated CMF is about 6% and the density profile of the interior is illustrated in Supplementary Figure 6. Such a small iron core would indicate a very low Fe/Si ratio (~0.32), lower than that of any known chondrites. Of course, there are compositional degeneracy and large observational uncertainty to be considered for a detailed model, but it illustrates the importance for an accurate reference mantle model because a different first-order question might be emphasized. Similarly, several relatively well characterized "hot" super-Earths (e.g., Kepler 10b, 36b, 80e, and 93b, CoRoT-7b, and HD-219134b) plot close to the Earth CMF curve clustered near 4 M_E (Fig. 3), implying a similar metal-silicate ratio as the Earth without considering the trade-off between mantle composition and CMF. On the other hand, the "cold" super-Earths (e.g., Kepler 186f, 62f, and 138c, and Gliese-667Cc) plotted close to the $MgSiO_3$ curve, may need to add a H_2O component in order to have a sizable iron core. Although large uncertainties in interpretation will remain without the improved mass and radius measurements and additional observations, the accurate density measurements of $MgSiO_3$ mantle from this study and Fe core by laser-driven compression[30] represent a significant advance from mineral physics towards interior models of exoplanets.

Understanding the melting temperature in deep mantle is important for the mantle dynamics and evolution of super-Earths. The observed high melting temperature of $MgSiO_3$ at great depths provides an upper bound for modeling deep mantle melting processes. It requires more energy to be retained from accretion to melt the mantle, therefore, influence the depth of magma ocean if global melting occurs at the end of planetary accretion. We recently investigated how the initial internal heat of the planet after formation scales with planet size[32]. Figure 3b shows the calculated initial temperatures at the core-mantle boundary (CMB) following accretion of the super-Earths, assuming simple $MgSiO_3$-mantle and Fe-core. Depending on the heat retention

Deleted: mantle[31]
Deleted: incompressible[32]
Deleted: phase[33]
Deleted: [29]
Formatted: Font: Italic
Deleted: -
Deleted:
Formatted: Font: Italic
Deleted: ,
Deleted: whereas
Deleted: when
Deleted: S5
Deleted: compression[28]
Deleted: The high
Deleted: has
Deleted: implications
Deleted: and influence the depth of magma ocean if global melting occurs at the end of planetary accretion.
Deleted: , and it
Deleted: .
Deleted: size[34]

efficiency (*f*) during accretion, the calculated CMB temperatures of the super-Earths (M_E >2) with an Earth-like f = 0.04 or higher, are sufficient to melt the entire mantle even with our high $MgSiO_3$ melting temperature (Fig. 3b). The calculation is based on a mantle potential temperature of 1725 K [37]. For a higher mantle potential temperature in the early history, it is expected that extensive mantle melting in super-Earths would be common following accretion. A similar conclusion was reached in recent thermal model of super-Earths[33].

The high melting temperature of $MgSiO_3$ at 500 GPa implies a high mantle solidus in super-Earths. If the mantle temperature is anywhere near its solidus, the core would be fully liquid and thermal convection or supersaturation of light elements in the liquid core such as MgO or SiO_2 is required to drive the dynamo[38-40]. Once the mantle and core cool down enough for the core to start to solidify, the mantle temperature would be very far below its solidus because the mantle solidus is a stronger function of planet mass than the mantle adiabat (Fig 2b). Therefore, the mantles of massive planets could be more viscous, resulted from the high melting temperature, which would reduce the core cooling rate. Earth's core is thought to be marginal for thermal convection today (i.e. the CMB heat flow is similar to the conductive requirement to drive convection)[41]. Compared to Earth, therefore, the cores of more massive planets tend to cool more slowly due to the increased mantle viscosity and have a higher core thermal conductivity, implying that thermal convection is less likely. Compositional convection in the core would also be less likely in massive planets because their cores initially start higher above the core liquidus[32] and a lot more cooling is required to begin core solidification, compared to Earth. In conclusion, massive rocky planets may be able to drive a dynamo early on when cooling is fast, then have no dynamo for billions of years while the interior is slowly cooling, and then possibly drive a compositional dynamo billions of years later.

Deleted: Earths[30]
Deleted: dynamo[35]
Deleted: [37]
Deleted: will
Deleted: will tend to
Deleted: based on homologous law
Deleted: will
Deleted: [38]
Deleted: will
Deleted: will
Deleted: will
Deleted: liquidus[34]

References:

1. Zerr, A. & Boehler, R. Melting of (Mg, Fe)SiO_3-perovskite to 625 Kilobars: indication of a high melting temperature in the lower mantle melting. *Science* **262**, 553–555 (1993).
2. Luo, S.-N., Akins, J. A., Ahrens, T. J ., & Asimow, P. D. Shock-compressed $MgSiO_3$ glass, enstatite, olivine, and quartz: Optical emission, temperatures, and melting, *J. Geophys. Res.* **109**, B05205 (2004).
3. Fratanduono, D. E. et al. Thermodynamic properties of $MgSiO_3$ at super-Earth mantle conditions. *Phys. Rev. B* **97**, 214105 (2018).
4. Ono, S., Kikegawa, T. & Ohishi, Y. Equation of state of $CaIrO_3$-type $MgSiO_3$ up to 144 GPa. *Am. Mineral*. **91**, 475–478 (2006).
5. Guignot, N., Andrault, D., Morard, G., Bolfan-Casanova, N. & Mezouar, M. Thermoelastic properties of post-perovskite phase $MgSiO_3$ determined experimentally at core–mantle boundary P–T conditions. *Earth Planet. Sci. Lett*. **256**, 162–168 (2007).
6. Komabayashi, T. et al. Simultaneous volume measurements of post-perovskite and perovskite in $MgSiO_3$ and their thermal equations of state. *Earth Planet. Sci. Lett*. **265**, 515–524 (2008).
7. Sakai, T., Dekura, H. & Hirao, N. Experimental and theoretical thermal equations of state of $MgSiO_3$ post-perovskite at multi-megabar pressures. *Sci. Rep.* **6**, 22652 (2016).

8. Tschauner, O. et al. Mineralogy. Discovery of bridgmanite, the most abundant mineral in Earth, in a shocked meteorite. *Science* **346**, 1100–1102 (2014).
9. Murakami, M., Hirose, K., Kawamura, K., Sata, N. & Ohishi, Y. Post-perovskite phase transition in $MgSiO_3$. *Science* **304**, 855–858 (2004).
10. Tange, Y., Kuwayama, Y., Irifune, T., Funakoshi, K. & Ohishi, Y. P-V-T equation of state of $MgSiO_3$ perovskite based on the MgO pressure scale: A comprehensive reference for mineralogy of the lower mantle. *J. Geophys. Res*. **117**, B06201 (2012).
11. Sun, N. et al. Phase transition and thermal equation of state of (Fe,Al)-bridgmanite and post-perovskite: Implication for the chemical heterogeneity at the lowermost mantle. *Earth Planet. Sci. Lett.* **490**, 161–169 (2018).
12. Deng, L., Gong, Z. & Fei, Y. Direct shock wave loading of $MgSiO_3$ perovskite to lower mantle conditions and its equation of state. *Phys. Earth Planet Inter.* **170**, 210-214 (2008).
13. Mosenfelder, J. L., Asimow, P. D., Frost, D. J., Rubie, D. C. & Ahrens, T. J. The $MgSiO_3$ system at high pressure: Thermodynamic properties of perovskite, postperovskite, and melt from global inversion of shock and static compression data. *J. Geophys. Res.* **114**, B01203 (2009).
14. Hinkel, N. R., Timmes, F. X., Young, P. A., Pagano, M. D. & Turnbull, M. C. Stellar abundances in the solar neighborhood: the Hypatia Catalog. *Astron. J.* **148**, 54–87 (2014).
15. Albareti, F. D. et al. The 13th data release of the Sloan Digital Sky Survey: First spectroscopic data from the SDSS-IV survey mapping nearby galaxies at Apache Point Observatory. *Astrophys. J.* **233** (suppl.), 25 (2017).
16. Duffy, T., Madhusudham, N. & Lee, K.K.M. Mineralogy of super-Earth planets. In *Treatise on Geophysics*, 2nd ed.; Elsevier: Oxford, UK, Volume **2**, pp. 149–178 (2015).
17. Root, S. et al. The principal Hugoniot of forsterite to 950 GPa. *Geophys. Res. Lett*. **45**, 3865–3872 (2018).
18. Millot, M. et al. Recreating giants impacts in the laboratory: Shock compression of $MgSiO_3$ bridgmanite to 14 Mbar. *Geophys. Res. Lett*. **47**, e2019GL085476 (2020).
19. Akins, J. A., Luo, S.-N., Asimow, P. D. & Ahrens, T. J. Shock-induced melting of $MgSiO_3$ perovskite and implications for melts in Earth's lowermost mantle. *Geophys. Res. Lett*. **31**, L14612 (2004).
20. Watt, J. P. & Ahrens, T. J. Shock wave equation of state of enstatite, *J. Geophys. Res*. **91***,* 7495-7503 (1986).
21. Morard, G. et al. In situ X-ray diffraction of silicate liquids and glasses under dynamic and static compression to megabar pressure. *Proc. Natl. Acad. Sci. U.S.A.* **117**, 11981-11988 (2020).
22. Hernandez, J. -A. et al. Direct observation of shock-induced disordering of enstatite below the melting temperature. *Geophys. Res. Lett*. **47**, e2020GL088887 (2020).
23. Knudson, M. D. & Desjarlais, M. P. Adiabatic release measurements in α-quartz between 300 and 1200 GPa: Characterization of α-quartz as a shock standard in the multimegabar regime. *Phys. Rev. B* **88**, 184107 (2013).
24. McCoy, C. A., Knudson, M. D. & Root, S. Absolute measurement of the Hugoniot and sound velocity of liquid copper at multimegabar pressures. *Phys. Rev. B* **96**, 174109 (2017).
25. Barker, L. M. & Hollenbach, R. E. Laser interferometer for measuring high velocities of any reflecting surface. *J. Appl. Phys*. **43**, 4669 (1972).

Formatted: Font: Italic
Formatted: Font: Italic
Deleted: 21
Deleted: 22
Deleted: 23

26. McCoy, C. A., Knudson, M. D. & Desjarlais, M. P. Sound velocity, shear modulus, and shock melting of beryllium along the Hugoniot. *Phys. Rev. B* **100**, 054107 (2019).
27. Oganov, A. R. & Ono, S. Theoretical and experimental evidence for a post-perovskite phase of $MgSiO_3$ in Earth's D" layer. *Nature* **430**, 445–448 (2004).
28. Belonoshko, A. B. et al. High-pressure melting of $MgSiO_3$. *Phys. Rev. Lett*. **94**, 195701 (2005).
29. Vinet, P., Rose, J., Ferrante, J., & Smith, J. Universal features of the equation of state of solids. *J. Phys. Cond. Matter* **1**, 1941–1963 (1989).
30. Smith, R. F. et al. Equation of state of iron under core conditions of large rocky exoplanets. *Nature Astronomy* **2**, 452–458 (2018).
31. Zeng, L. & Sasselov, D. A. Detailed model grid for solid planets from 0.1 through 100 Earth masses. *Publ. Astron. Soc. Pac.* **125**, 227–239 (2013).
32. Boujibar, A., Driscoll, P., & Fei, Y. Super-Earth internal structures and initial thermal states. *J. Geophys. Res. Planets* **125**, e2019JE006124 (2020).
33. Stixrude, L. Melting in Super-Earths. *Philos. Trans. R. Soc. A* **372**, 20130076 (2014).
34. Coppari, F. et al. X-ray diffraction study of a new phase of MgO at exo-planet interior pressures. *Nat. Geosci.* **6**, 926–929 (2013).
35. Millot, M. et al. Shock compression of stishovite and melting of silica at planetary interior conditions. *Science* **347**, 418-420 (2015).
36. Kuwayama, Y., Hirose, K., Sata, N. & Ohishi, Y. The pyrite-type high-pressure form of silica. *Science* **309,** 923–925 (2005).
37. Herzberg, C. et al. Temperatures in ambient mantle and plumes: Constraints from basalts, picrites, and komatiites. *Geochem. Geophys. Geosyst.*, **8**, Q02006 (2007).
38. O'Rourke, J. G. & Stevenson, D. J. Powering Earth's dynamo with magnesium precipitation from the core. *Nature* **529**, 387–389 (2016).
39. Badro, J., Siebert, J. & Nimmo, F. An early geodynamo driven by exsolution of mantle components from Earth's core. *Nature* **536**, 326–328 (2016).
40. Hirose, K. et al. Crystallization of silicon dioxide and compositional evolution of the Earth's core. *Nature* **543**, 99–102 (2017).
41. Driscoll, P. E. & Du, Z. Geodynamo conductivity limits. *Geophys. Res. Lett.*, **46**, 7982–7989 (2019).

Deleted: 24
Deleted: 25
Deleted: 26
Deleted: 27
Deleted: 28
Deleted: 29
Moved (insertion) [1]
Deleted: 4
Deleted: 30
Deleted: 31
Deleted: 32
Deleted: 33
Moved up [1]: 34. Boujibar, A., Driscoll, P., & Fei, Y. Super-Earth internal structures and initial thermal states. *J. Geophys. Res. Planets* **125**, e2019JE006124 (2020).
Formatted: Font: Italic
Formatted: Font: Bold
Deleted: 35
Deleted: 36
Deleted: 37
Deleted: 38

Table 1. Hugoniot data for $MgSiO_3$ using bridgmanite as starting material.

**Sample**	**flyer**	u_f	u_p	U_s	**P**	ρ	V_P	V_B
		km/s	km/s	km/s	GPa	g/cm^3	km/s	km/s
Z3268-N2	Al	12.45(0.04)	5.11(0.08)	14.20(0.15)	297(5)	6.40(0.10)		
Z3268-N4	Al	12.35(0.04)	5.04(0.07)	14.32(0.13)	296(4)	6.32(0.09)		
**Z3268-ave**			**5.07(0.05)**	**14.26(0.10)**	**296(4)**	**6.36(0.07)**		
Z3203-N4	Cu	9.51(0.04)	5.55(0.05)	15.32(0.17)	349(5)	6.43(0.09)		
Z3203-N6	Cu	9.53(0.04)	5.59(0.06)	15.05(0.27)	345(5)	6.53(0.14)		
Z3203-S4	Cu	9.63(0.04)	5.68(0.06)	14.87(0.34)	347(6)	6.64(0.18)		
Z3203-S6	Cu	9.64(0.04)	5.67(0.06)	15.11(0.31)	351(6)	6.56(0.16)		
**Z3203-ave**			**5.62(0.03**	**15.13(0.13)**	**348(3)**	**6.52(0.07)**	**19.38(1.34)**	**14.12(1.90)**
Z3116-N4	Cu	10.90(0.04)	6.43(0.07)	16.01(0.41)	423(8)	6.85(0.21)		
Z3116-N6	Cu	10.91(0.04)	6.46(0.06)	15.83(0.38)	419(8)	6.93(0.20)		
Z3116-S4	Cu	10.90(0.04)	6.47(0.06)	15.73(0.37)	417(7)	6.97(0.20)		
Z3116-S6	Cu	10.93(0.04)	6.43(0.06)	16.17(0.30)	427(7)	6.81(0.15)		
**Z3116-ave**			**6.45(0.03)**	**15.95(0.18)**	**422(4)**	**6.88(0.09)**	**20.15(2.35)**	**14.86(0.85)**
Z3029-N4	Cu	12.20(0.04)	7.24(0.07)	16.86(0.19)	500(6)	7.19(0.11)	18.28(1.94)	
Z3029-N6	Cu	12.25(0.04)	7.31(0.08)	16.58(0.51)	497(11)	7.34(0.28)	17.95(2.02)	
Z3029-S4	Cu	12.21(0.04)	7.25(0.09)	16.82(0.77)	500(15)	7.23(0.40)	17.68(1.25)	
Z3029-S6	Cu	12.29(0.04)	7.30(0.10)	16.86(1.00)	505(19)	7.26(0.51)	16.88(1.56)	
**Z3029-ave**			**7.27(0.04)**	**16.80(0.21)**	**500(5)**	**7.24(0.12)**	**17.65(0.82)**	**15.39(1.89)**
Z3011-N4	Cu	15.24(0.04)	9.01(0.08)	19.65(0.31)	726(10)	7.58(0.16)		
Z3011-N6	Cu	15.24(0.04)	9.10(0.08)	19.03(0.18)	710(8)	7.86(0.12)		
Z3011-S4	Cu	15.24(0.04)	9.08(0.09)	19.19(0.50)	714(13)	7.79(0.26)		
Z3011-S6	Cu	15.23(0.04)	9.08(0.08)	19.14(0.26)	712(10)	7.80(0.15)		
**Z3011-ave**			**9.07(0.04)**	**19.22(0.14)**	**715(5)**	**7.76(0.08)**		**17.35(1.10)**
Z3250-S2	Cu	16.96(0.04)	10.11(0.09)	20.50(0.13)	850(9)	8.09(0.11)		
Z3250-S4	Cu	17.02(0.04)	10.15(0.09)	20.54(0.15)	855(10)	8.11(0.11)		
Z3250-S6	Cu	17.04(0.04)	10.13(0.09)	20.79(0.23)	864(11)	8.00(0.14)		
**Z3250S-ave**			**10.13(0.05)**	**20.58(0.09)**	**856(6)**	**8.07(0.07)**		**18.07(0.78)**
Z3250-N2	Cu	17.68(0.04)	10.53(0.09)	21.18(0.12)	914(10)	8.16(0.10)		
Z3250-N4	Cu	17.64(0.04)	10.53(0.09)	20.98(0.17)	906(10)	8.23(0.12)		
Z3250-N6	Cu	17.62(0.04)	10.51(0.09)	21.06(0.21)	907(11)	8.18(0.14)		
**Z3250N-ave**			**10.52(0.05)**	**21.09(0.09)**	**909(6)**	**8.19(0.07)**		**20.01(1.24)**
Z3321-S2	Al	29.29(0.04)	12.52(0.17)	23.43(0.45)	1202(22)	8.81(0.27)		
Z3321-S4	Al	29.26(0.04)	12.41(0.17)	23.82(0.40)	1212(21)	8.56(0.24)		
**Z3321S-ave**			**12.47(0.12)**	**23.64(0.30)**	**1207(15)**	**8.68(0.18)**		
Z3321-N2	Al	North 2 Sample Compromised						
Z3321-N4	Al	30.00(0.04)	**12.83(0.19)**	**23.86(0.15)**	**1254(29)**	**8.87(0.23)**		

METHODS

Experiments: The dynamic compression experiments were perfomed at the Sandia National Laboratory Z machine[42] that is capable of generating magnetically-accelerating flyer plates with impact velocities up to 42 km/s. We used solid aluminum or composite copper/aluminum flyer plates as impactors for the plate-impact experiments. The copper/aluminum flyer plate consists of a nominally 250 micron copper layer supported by a 0.9 mm aluminum layer precisely fabricated by diamond-turning[24,26]. The target was assembled in the coaxial load geometry, consisting of a north and a south sample panel with 7 sample holders on each panel (Supplementary Fig. 1). The pre-synthesized polycrystalline $MgSiO_3$-bridgmanite samples were nominally 2.3-mm disks with variable thicknesses (0.3659 - 0.9339 mm), backed by Z-cut α-quartz windows. The bridgmanite samples are not transparent. Adjacent samples were transparent quartz or diamond/quartz windows which enabled observation of the flyer plate and determination of the impact time at the sample.

Syntheses of the $MgSiO_3$-bridgmanite samples were carried out in the multi-anvil press at the Carnegie Institution for Science[43]. The high-pressure assembly used in this study is similar to that of previous study[12], but the tungsten-carbide cubes were replaced by 25.4-mm sintered-diamond cubes. Cylinderical bridgmanite samples with diameters of ~2.3-mm were synthesized at 25 GPa and 1673 K, using the same sintered $MgSiO_3$ enstatite as starting material for all the syntheses. The recovered samples were polished into disks with parallel surfaces. More than 34 bridgmanite samples were prepared for the shock wave experiments. The chemical compositions of the recovered bridgmanite were confirmed by electron microprobe analyses, with representative composition of MgO (40.70±0.39 wt%) and SiO_2 (59.85±0.31wt%), consistent with the $MgSiO_3$ chemical formula. The denisty of the crystal is 4.103±0.002 g/cm^3, determined from its unit cell parameters (a = 4.7763±0.0006 Å, b = 4.9314±0.0006 Å, and c = 6.8992±0.0010 Å) by X-ray diffraction technique. Densities of selected samples were also measured by the Archimedes method, with an average value close to 4.10 g/cm^3, but the uncertainty is relatively large (~1 %) because of the small sample size.

For symmetric-target plate-impact experiments, the north and south flyer plates impacted the corresponding targets (the north and south sample panels) at the same velocity (Supplementary Fig. 1). The flyers were launched at impact velocities between 9.5 and 30.0 km/s (Table 1). For the two highest pressure experiments (Z3250 and Z3321), the impact velocities were intentionally slightly different on the north and south panels and the data were analyzed separately for the two panels. The flyer velocity and impact time at the sample were measured through adjacent transparent windows by multifiber VISAR probes[25]. Corrections are applied for dynamic flyer tilt (interpolation) and measured flight gap differences from sample to sample due to imperfect assembly/mounting of the target panel. The measured impact time difference from the top to the bottom of the panel is typically ~1-3 ns. Part of this is due to dynamic flyer bowing/tilt during acceleration and part due to variations in the flight gap distance between the initial flyer position and the samples (assembly and alignment precision). The flight gap correction is typically on the order of ~0.5 ns (resulting from ~10 micron differences in flight gap). These corrections are small, but important, and were applied for every bridgmanite Hugoniot measurement presented using the unique above/below measured impact times and flight gaps.

Deleted: machine[39]

Deleted: turning[22]

Deleted: [24]

Deleted: S

Deleted: Science[40]

Deleted: S

Deleted: probes[23]

Additional velocity histories were recorded in 3 channels for each bridgmanite sample with three different fringe sensitivities (typically 405, 1057, 2290 m/s/fringe in vacuum). The bridgmanite shock velocity (U_s) was determined from the transit time for the shock to traverse the sample. The uncertainties in shock velocity are less than 2%. The bridgmanite particle velocity (u_p) and associated uncertainty were determined with impedance matching in a Monte Carlo technique which varied the equation of state of the flyer by ±3% and the flyer velocity, bridgmanite shock velocity, and bridgmanite initial density within the stated uncertainties; the mean and standard deviation of the particle velocity are reported in Table 1. The copper equation of state used was SESAME 3325, and the aluminum equation of state used was SESAME 3700[44]. The Hugoniot in both the copper and aluminum equation of state models utilized for impedance matching the bridgmanite Hugoniot have been validated by multiple studies[24,45]. For several experiments (e.g. Z3011, Z3029,and Z3116), copper and aluminun samples were also mounted as standards for additional validation.

Deleted: 3700[41]

Deleted: studies[22]

Deleted: [42]

Sound velocity analysis: The sound velocity was determined using the overtaking rarefaction method with quartz windows. In the overtaking rarefaction method, the backwards-facing shock in the composite flyer reaches the Cu/Al interface after propagating a known distance. Upon reaching the interface, the shock pressure releases due to the large density discontinuity, resulting in a rarefaction wave that propagates through the flyer and sample, eventually overtaking the shock front. This method has previously been used to measure the sound velocity in shocked copper[24] and beryllium[26]. The use of quartz windows decreases the uncertainty in the overtake time and enables additional analysis of the wave profile. For all shots, the shock transmitted from the Bridgmanite into the quartz window was of sufficient strength to melt and partially ionize the quartz, generating a reflecting shock front which was observed with VISAR. Upon overtake of the rarefaction wave, the shock velocity begins to decrease and is clearly visible in the wave profile.

Deleted: copper[22]

Deleted: beryllium[24]

The bridgmanite sound velocity was analyzed using both multi-sample and single-sample techniques to determine the overtake time and thickness. Because the single-sample technique requires an assumption of a scaled sound velocity for waves propagating through released material[22], this technique was used for the shots where velocity could not be determined by the multi-sample method. In both techniques, the wave profile was discretized using linear fits to the constant velocity plateau, longitudinal release (if present) and bulk release (Supplementary Fig. 4). The plateau fit and release fits were extrapolated to find their intersection, which determined the overtake time in the window for a particular sample.

Deleted: S3

The multi-sample technique leveraged the different bridgmanite sample thicknesses fielded to relate the window overtake time (t_{WO}) with the sample thickness. The sample thickness was linearly fit as a function of t_{WO} and extrapolated to t_{WO} = 0. This determined the depth (d_∞) and time (t_∞) at which overtake would occur in an infinitely thick bridgmanite sample. The bridgmanite sound velocity is given as

$$C_L = \frac{d_\infty}{t_\infty - t_L} \qquad (1)$$

where $t_L = t_I + \frac{d_f}{U_S^{Cu}} + \frac{d_f}{C_L^{Cu}}$ is the time when the rarefaction wave enters the bridgmanite, t_I is the impact time, d_f is the thickness of the copper layer on the flyer, and U_S^{Cu} and C_L^{Cu} are the copper shock velocity and Lagrangian sound velocity, respectively. This technique was used to determine the bulk wave speed in both solid and liquid bridgmanite.

The longitudinal wave speeds in solid bridgmanite were determined by the single-sample analysis. The wave profiles from two low-pressure shots showed release from a solid state (Supplementary Figs. 4b and 4c). In the single-sample technique, the overtaking wave propagates through both shocked and partially-released bridgmanite prior to breaking out into the quartz window. The amount of shocked and partially-released material that the overtaking wave traverses is determined by solving the system of equations which define the bridgmanite characteristics shown in Supplementary Figure 3. The three waves which impact the overtake are the overtaking and window rarefactions (dashed red lines) and partially-released rarefaction (dashed-dotted purple line). This system defines the characteristic intersection (x_{int}, t_{int}) and Lagrangian sound velocity as

$$t_{int} = \frac{S_{C_L} t_{IO} + t_q}{1 + S_{C_L}}, \qquad (2)$$

$$C_L = \frac{d_b}{2t_{int} - \left(t_q + t_L\right)}, \qquad (3)$$

$$x_{int} = C_L \left(t_{int} - t_L\right), \qquad (4)$$

where S_{C_L} is the sound velocity scale factor for partially-released bridgmanite, t_{IO} is the overtake time at the bridgmanite-quartz interface, t_q is the time of shock breakout into the quartz window, and d_b is the bridgmanite sample thickness.

Single-sample analysis of the three shots where the wave profiles exhibited a simple release determined $S_{C_L} = 0.89 \pm 0.07$. Copper and quartz simulations[24,26] have previously indicated that the scale factor is approximately pressure-independent so the constant value remains valid for the low-pressure data even though it was calculated from the three highest-pressure shots. The overtake time was determined by using a characteristics method to transform the quartz shock velocity into an interface particle velocity[46] and fitting the plateau and release regions of the particle velocity. This method used the most recent quartz Hugoniot and release models[47] to define the quartz sound velocity as a function of the measured shock velocity.

Because the single-sample analysis only considers first-order characteristic interactions, it is only valid for the initial release wave and cannot be used to determine the bulk sound speed in a solid. Extension to the bulk speed would require additional scale factors for second-order interactions which are unable to be constrained experimentally.

On shot Z3203, the wave profile indicates that a three-stage release occurred (Supplementary Fig. 4c), with the initial release attributed to an edge rarefaction impinging on the shock front in the center of the target prior to overtake of the longitudinal wave. The edge wave was also observed in the shot Z3029 (Supplementary Fig. 5). As the bridgmanite samples were irregularly shaped, the edge wave could not be used for sound speed analysis and

Deleted: S3b
Deleted: S3c
Deleted: .
Deleted: S2
Deleted: simulations[22]
Deleted: [24]
Deleted: velocity[43]
Deleted: models[44]
Deleted: S3c
Deleted: S4

modification to the overtaking wave analysis was required. From the behavior of the shock front and overtaking rarefaction in the quartz window shown in Supplementary Figure 3, the time at which the rarefaction overtakes the edge wave accurately represents the overtake that would occur without an edge wave present (Δt < 0.1 ns). Fitting to the plateau generates an overtake time ~1.5 ns earlier, significantly increasing the measured sound velocity. Because the edge wave propagates inward with nominally constant velocity, the expected time where the edge wave reaches the center of the bridgmanite/quartz interface is approximately the same as when it reaches the center of the shock front. To account for this, the quartz window analysis was modified such that at each time-step, the characteristic sound velocity was adjusted according to the edge wave pressure at the shock front. This analysis is unable to account for edge waves propagating at the longitudinal velocity in the bridgmanite impacting the overtaking wave, and hence should be considered an upper bound on the sound speed. Similarly, the bulk velocity calculated with the multi-sample analysis should be considered an upper bound due to unaccounted edge wave effects

Uncertainties were calculated using a Monte Carlo method with 10^5 iterations. For the overtake times, plateau and release regions of interest were defined and the fit was calculated with variable-length intervals of 10-15 ns for the plateau and 5-10 ns for the release. For experiments where a release region was shorter than 10 ns the fit interval range was 0.5-1x the region of interest. Typical uncertainties in overtake times were <2 ns, and the overtake transit time was the dominant uncertainty for the bulk of these experiments. The thickness of the copper layer on the flyer was calculated from the copper and aluminum density and measurements of the flyer mass and dimensions. The overall flyer thickness was measured to <0.5 µm using an enhanced dual confocal microscope[48], and lateral dimensions and mass uncertainties were ~10 µm and ~0.1 mg, respectively. This produced a copper thickness uncertainty of ~1 µm. On Z3203, accounting for the edge wave introduced a systematic error of ~3.5% to both the longitudinal and bulk sound speed; this produced total uncertainties of ~11% and ~13% respectively.

Computational methods: We performed a series of density functional theory (DFT) calculations for the bridgmanite, post-perovskite, and liquid phases of $MgSiO_3$. All calculations were performed using the VASP version 5.4 code, an implementation of Kohn-Sham DFT using a plane wave basis, projector-augmented-wave pseudopotentials, and periodic boundary conditions[49-51]. The exchange and correlation contributions to the energy were computed using the Perdew, Burke, Ernzerhof (PBE) generalized gradient approximation[52]. The VASP projector augmented wave (PAW) pseudopotentials were used with a plane wave energy cutoff of 800 eV to ensure convergence of the stress tensor. To accurately capture the semicore excitations in magnesium at high temperature, we used a magnesium pseudopotential with a $2p^6 3s^2$ valence configuration, while that of silicon and oxygen were $3s^2 3p^2$ and $2s^2 2p^4$, respectively. All calculations used 160 atom supercells for the bridgmanite, post-perovskite, and liquid phases.

First, the equations of state (EOS) for the static lattice were obtained for bridgmanite and post-perovskite from a Vinet EOS[29] fit to the total energy of the lattice computed for a series of densities. A 6x6x6 k-point grid was found to yield converged values of the energy and stress tensor. For each density, the crystal geometry was optimized until the average force per atom was less than 0.01 meV/atom, but the overall crystal symmetry was constrained to be orthorhombic.

Deleted: .
Deleted: S2
Deleted: microscope[45]
Deleted: conditions[46]
Deleted: [48]
Deleted: approximation[49]
Deleted: EOS[27]

Second, the principal Hugoniot of bridgmanite was computed from a set of DFTMD calculations. Our DFTMD calculations follow a similar approach as in previous work[17,53]. The Brillouin zone was sampled at a single *k*. The electronic occupations were populated according to the Mermin finite temperature formulation of DFT[54]. The reference state for the Hugoniot calculations was obtained from the energy and pressure of a well-equilibrated DFTMD calculation of bridgmanite at 300K with a density of 4.10 g/cm^3. Hugoniot states were then computed from linear interpolation of the temperature dependence of the energy and pressure for a set of isochores such that the Rankine-Hugoniot relation was satisfied:

Deleted: 50

Deleted: DFT[51]

$$E - E_0 = \frac{1}{2}(P + P_0)(V - V_0) \tag{5}$$

With E, P, V denoting the energy, pressure, and volume of the Hugoniot state, respectively, and the subscript indicating values for the reference state of bridgmanite. For bridgmanite and post-perovskite, the lattice parameter ratios for the high-temperature DFTMD calculations were unchanged from their optimized 0 K values. Results of the DFTMD calculations for $MgSiO_3$ post-perovskite and the liquid phase are shown in Figure 1.

With the Hugoniot states in hand, we then computed the bulk sound velocity in the liquid phase. For a point on the Hugoniot, the adiabatic bulk modulus is given by:

$$K_S = (P - P_0)\frac{\gamma}{2} + \rho\left(\frac{dP}{d\rho}\right)\Big|_H \left[1 + \left(1 - \frac{\rho}{\rho_0}\right)\frac{\gamma}{2}\right] \tag{6}$$

With K_S the adiabatic bulk modulus, $P - P_0$ the pressure difference between the Hugoniot and reference state, γ the Grüneisen parameter, ρ the density on the Hugoniot, and $\left(\frac{dP}{d\rho}\right)\Big|_H$ the derivative of the Hugoniot pressure with respect to the Hugoniot density, evaluated at the point on the Hugoniot in question. The only term which requires some care is the estimation of the pressure derivative. A typical Hugoniot calculation is done on a coarse density grid, which makes a finite difference estimation of the derivative suspect. We found that fitting the entire P, ρ Hugoniot curve with a cubic polynomial to provide a more robust, and analytic, estimate of this term. We call this the "derivative method".

The resulting estimates of the bulk modulus were cross checked by performing a set of follow-up calculations. It is well known that compression of a hydrodynamic material due to an infinitely weak shock is isentropic. Therefore, the isentropic bulk modulus can be estimated directly according to the definition:

$$K_S = \rho\left(\frac{\partial P}{\partial \rho}\right)_S \cong \frac{P_2 - P_1}{1 - \frac{\rho_1}{\rho_2}} \tag{7}$$

where a pair of points (P_1, ρ_1) and (P_2, ρ_2) provide that the latter point is on the Hugoniot for which the former point is the reference state. In other words, taking (P_1, ρ_1) as a reference state, one satisfies the Rankine-Hugoniot relations for a density ρ_2 a small distance away from ρ_1. We call this the "release method" because in the experimental literature such states are sometimes called *release* states, that describe isentropic decompression of a material after the

shock wave has passed through. The results from the "release method" are consistent with those from the "derivative method". The bulk sound velocities calculated from the bulk modulus and density as a function of pressure are listed in Supplementary Table 1. We also provided the statistical uncertainties in the Hugoniot states which give the statistical uncertainties in the underlying $E(V,T)$, and $P(V,T)$ data from the DFTMD calculations. The small errors in pressure indicate that the underlying data is well equilibrated and therefore representative of the system in equilibrium. Comparison of the experimental and theoretical estimates are shown in the main text in Figure 2a.

Deleted: S

Thermal equation of state: The pressure(P)-density(ρ)-temperature(T) relation is calculated by

$$P(\rho,T) = P(\rho,300\mathrm{K}) + P_{\mathrm{th}} \quad (8)$$

where $P(\rho,300\mathrm{K})$ is calculated by the Vinet equation

$$P(\rho,300\mathrm{K}) = 3K_0\left(\frac{\rho_0}{\rho}\right)^{-2/3}\left[1-\left(\frac{\rho_0}{\rho}\right)^{1/3}\right]\exp\left\{\frac{3}{2}(K_0'-1)\left[1-\left(\frac{\rho_0}{\rho}\right)^{1/3}\right]\right\} \quad (9)$$

The thermal pressure (P_{th}) is expressed by the Mie-Grüneisen relation:

$$P_{th} = \frac{\gamma}{V}[E(T,\theta_D) - E(300K,\theta_D)] \quad (10)$$

with the Grüneisen parameter, $\gamma = \gamma_0(\rho_0/\rho)^q$, and the Debye temperature, $\theta_D = \theta_{D0}\exp[(\gamma_0-\gamma)/q]$. The harmonic internal energy $E(T,\theta_D)$ is calculated from the Debye model[55].

Deleted: model[52]

The thermal equation of state with the optimized parameters reproduces the static room-temperature compression data[5-7] and the Hugoniot measurements up to 500 GPa and 9000 K (Fig. 1). Using these new parameters, we calculated the internal structure of super-Earths up to 10 M_E, following the same procedure described in our recent paper[32]. Supplementary Figure 6 shows representative density profiles super-Earth with 4 M_E and the exoplanet 55 Cnc e, with a mantle potential temperature of 1725 K.

Deleted: paper[34]

Deleted: S5

In order to estimate the initial temperatures at the core-mantle boundary (CMB) following super-Earth accretion, we calculated temperature as a function of mass from the accretional heat and compression heat[32]. Supplementary Figure 7 shows the contribution of the gravitational energy that is retained from accretion, ΔT_G, with an efficiency factor $f = 0.06$ and an Earth-like $f = 0.04$, and the mantle adiabat, ΔT_{ad}, with a mantle potential temperature of 1725 K. The ΔT_G has a stronger mass dependence, comparable to previous model[33]. The mantle potential temperature could be much higher immediately after accretion that would make global magma ocean in super-Earths common.

Deleted: heat[34]

Deleted: S6

Deleted: model[30]

Additional references:

42. Matzen, M. et al. Pulsed-power-driven high energy density physics and inertial confinement fusion research. *Phys. Plasmas* **12**, 055503 (2005).

Deleted: 39

43. Bertka, C. M. & Fei, Y. Mineralogy of the Martian interior up to core–mantle boundary pressures. *J. Geophys. Res*. **102**, 5251–5264 (1997).
Deleted: 40
44. Kerley, G. I. Theoretical equation of state for aluminum. *Int. J. Impact Engng*. **5**, 441-449 (1987).
Deleted: 41
45. Knudson et al. Near-absolute Hugoniot measurements in aluminum to 500 GPa using a magnetically accelerated flyer plate technique. J. Appl. Phys. 94, 4420-4431 (2003).
Deleted: 42
46. McCoy, C. A. & Knudson, M. D. Lagrangian technique to calculate window interface velocity from shock velocity measurements: Application for quartz windows. *J. Appl. Phys*. **122**, 085901 (2017).
Deleted: 43
47. Desjarlais, M. P., Knudson, M. D. & Cochrane, K. R. Extension of the Hugoniot and analytical release model of α-quartz to 0.2–3 TPa. *J. Appl. Phys*. **122**, 035903 (2017).
Deleted: 44
48. Tomlinson, K. et al. Enhanced dual confocal measurement system. *Fusion Sci. Tech.* **73**, 139-148 (2018).
Deleted: 45
49. Kresse, G. & Hafner, J. Ab initio molecular dynamics for liquid metals. *Phys. Rev. B* **47,** 558-561 (1993).
Deleted: 46
50. Kresse, G. & Hafner, J. Ab initio molecular-dynamics simulation of the liquid-metal-amorphous-semiconductor transition in germanium. *Phys. Rev. B* **49,** 14251-14269 (1994)
Deleted: 47
51. Kresse, G. & Furthmüller, J. Efficiency of ab-initio total energy calculations for metals and semiconductors using a plane-wave basis set. *Comp. Mat. Sci*. **6**, 15-50 (1996).
Deleted: 48
52. Perdew, J. P., Burke, K. & Ernzerhof, M. Generalized gradient approximation made simple. *Phys. Rev. Lett*. **77**, 3865-3868 (1996).
Deleted: 49
53. Root, S., Townsend, J. P. & Knudson, M. D. Shock compression of fused silica: An impedance matching standard. *J. Appl. Phys*. **126**, 165901 (2019).
Deleted: 50
54. Mermin, N. D. Thermal Properties of the inhomogeneous electron gas. *Phys. Rev*. **137**, A1441-A1443 (1965).
Deleted: 51
55. Fei, Y., Mao, H. K., Shu, J. & Hu, J. P-V-T Equation of State of Magnesiowüstite ($Mg_{0.6}Fe_{0.4}$)O. *Phys. Chem. Miner*. **18**, 416-422 (1992).
Deleted: 52

Acknowledgements:
The project is made possible by the Z Fundmental Science Program. We thank the teams at Sandia National Laboratories for target fabrication and the operation of the Z facility. The project is partially supported by Carnegie Venture grant and NSF grant (EAR-1619868 to YF). We thank Marcus Knudson for coordination of the experimental conditions, Xiaojun Hu for preparation of

the sintered enstatite starting material, and Jing Yang for sample characterization. This paper describes objective technical results and analysis. Any subjective views or opinions that might be expressed in the paper do not necessarily represent the views of the U.S. Department of Energy or the United States Government. Sandia National Laboratories is a multimission laboratory managed and operated by National Technology & Engineering Solutions of Sandia, LLC, a wholly owned subsidiary of Honeywell International Inc., for the U.S. Department of Energy's National Nuclear Security Administration under contract DE-NA0003525.

Author contributions

YF proposed and directed the project. CTS, MDF, and YF designed and performed the experiments. CTS, CAM, and YF did data analysis. JPT and LS perforned calculations. AB, PD, and YF modeled internal structure of super-Earth.YF wrote the manuscript and everyone contributed to the draft.

Supplementary Materials:

Supplementary Table 1. Calculated Hugoniot data, bulk modulus, and bulk sound velocity.

	Bridgmanite		Post-perovskite		Liquid			
ρ, g/cc	T_H, K	P, GPa	T_H, K	P, GPa	T_H, K	P, GPa	K_S, GPa	V_B, km/s
4.50	356(93)	40.0(0.1)						
5.00	610(82)	90.0(0.1)	482(84)	81.6(0.1)				
5.50	1333(88)	156.3(0.1)	1131(104)	144.4(0.1)				
6.00	2813(95)	242.5(0.1)	2538(83)	227.2(0.1)				
6.50	5337(88)	352.4(0.1)	5104(82)	334.2(0.1)	2223(166)	319.1(0.2)	1077(5)	12.87(0.02)
7.00	9019(92)	490.1(0.2)	8492(77)	469.2(0.2)	5505(89)	450.4(0.2)	1517(10)	14.72(0.06)
7.50			13272(311)	636(2)	9389(92)	611.0(0.3)	1861(17)	15.75(0.08)
8.00					14202(131)	812(1)	2314(47)	17.0(0.2)
8.50					21036(100)	1056(1)	2892(36)	18.4(0.2)
9.00					30474(95)	1350(1)	3492(91)	19.7(0.3)
9.50					42250(96)	1695(2)		

Note: Bridgmanite reference state for the calculations: E_0 = -33.072 MJ/kg, ρ_0 = 4.10 g/cc, P_0 = 10.302 GPa, and T_0 = 299.977 K

Deleted: s

Formatted: Font: Italic

Formatted: Subscript

Formatted: Subscript

Formatted: Font: Italic

Formatted: Subscript

Formatted: Font: Italic

Formatted: Subscript

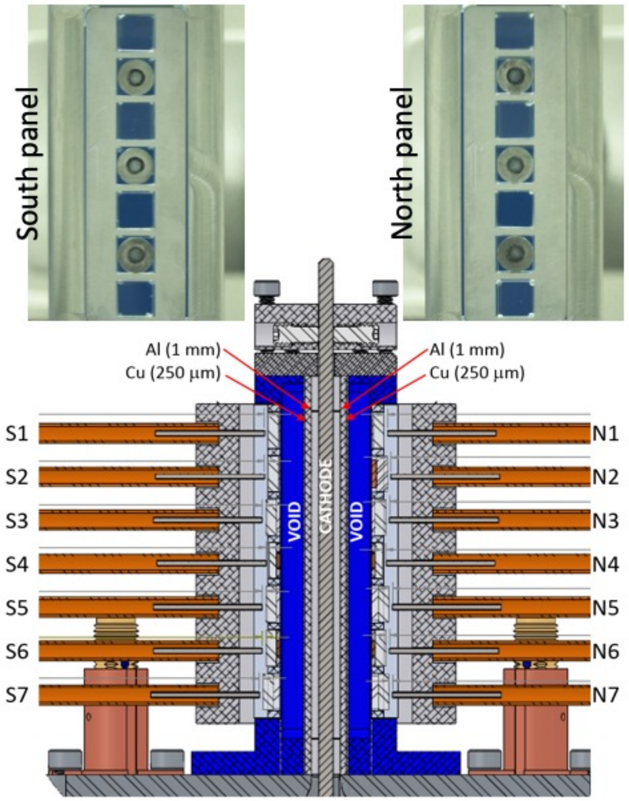

Supplementary Fig. 1. **Representative target configuration (Z3250) of the coaxial load geometry.** Labels S1 – S7 and N1 – N7 correspond to the VISAR probes as well as to the samples monitored. The bridgmanite samples are located at S2, S4, and S6 on the south panel and at N2, N4, and N6 on the north panel. The Cu/Al flyer separated from the cathode stalk at the center by A-K gaps (anode-cathode gap distances) in both sides. When firing the Z machine, the huge magnetic field in the A-K gaps generated by the stored energy from the capacitor banks drives the flyers to impact the target.

Deleted: S

Formatted: Font: Bold

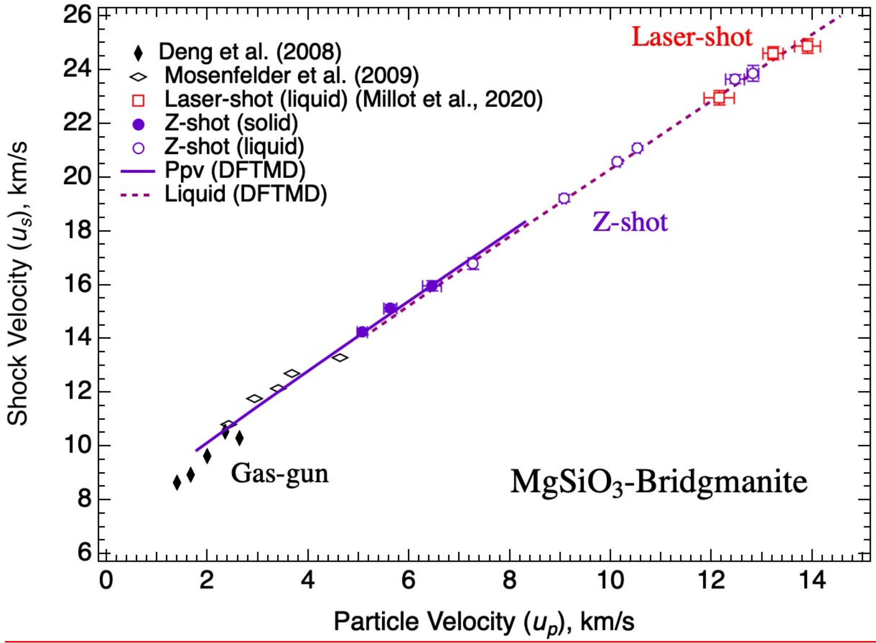

Supplementary Fig. 2. **Shock velocity U_S versus particle velocity u_p for $MgSIO_3$-bridgmanite starting material.** Data from this study (purple circles) are compared with liquid $MgSiO_3$ data by the laser-driven shock (red open squares)[18], and solid $MgSiO_3$ data (black solid diamonds[12] and open diamonds[13]) by gas-gun method. The purple solid and dashed lines are the calculated results for post-perovskite and liquid, respectively, by DFTMD simulations.

Formatted: Font: Bold
Formatted: Font: Bold, Subscript
Formatted: Font: Bold
Formatted: Font: Bold, Subscript
Formatted: Font: Bold
Formatted: Font: Bold, Subscript
Formatted: Font: Bold

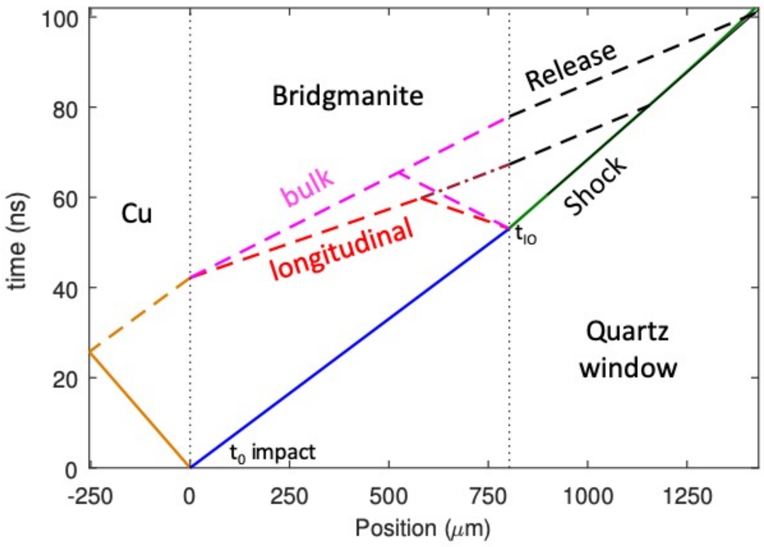

Supplementary Fig. 3. **Wave interactions in *x-t* diagram for overtaking wave analysis in solid.** The bridgmanite sound velocity was determined from the overtake time of rarefaction waves (dashed lines) with the shock front (solid lines). When the rarefaction wave from the copper/aluminum interface (orange) reaches the bridgmanite interface, it splits into longitudinal (red) and bulk (pink) overtaking waves. These waves are transmitted into the quartz window (black) where the overtake the quartz shock front (green). In the single-sample analysis, the overtaking wave interacts with a rarefaction wave from the bridgmanite/quartz interface and propagates at a reduced sound velocity (dashed-dotted purple line) which is incorporated into the analysis. The edge wave in the quartz window decreased the shock velocity such that the true overtake time is later than that at the edge wave by ~0.1 ns and ~1.5 ns for the longitudinal and bulk waves, respectively. Note that no interactions between the longitudinal and bulk waves in the bridgmanite were accounted for, so the extracted sound velocity should be considered an upper bound.

Deleted: S2

Formatted: Font: Bold

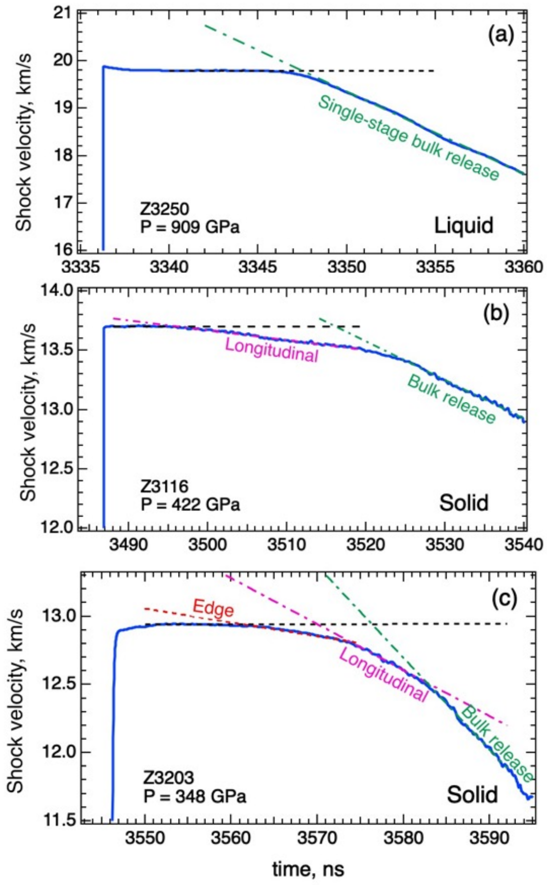

Supplementary Fig. 4. **Representative wave profiles for bridgmanite shocked into the liquid (a) and solid (b and c) phases. (a)** In the liquid phase, the bridgmanite shock velocity (solid blue line) exhibits a single-stage release that can be fit as a constant-velocity plateau (dashed black line) and bulk release (dashed-dotted green line). The intersection of these fits determines the overtake time for a given sample. **(b)** Shots with peak pressures below melting show a longitudinal release (dashed-dotted purple line) followed by a bulk release (dashed-dotted green line). **(c)** For the shot Z3203, an edge wave (dashed red line) reached the center of the target prior to the rarefaction wave overtaking the shock front, in addition to the longitudinal (dashed-dotted purple line) and bulk release (dashed-dotted green line).

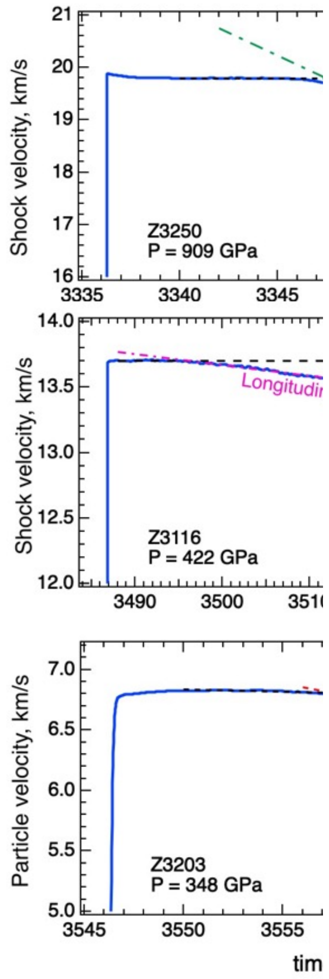

Deleted:

Deleted: S3

Formatted: Font: Bold

Formatted: Font: Bold

Formatted: Font: Bold

Formatted: Font: Bold

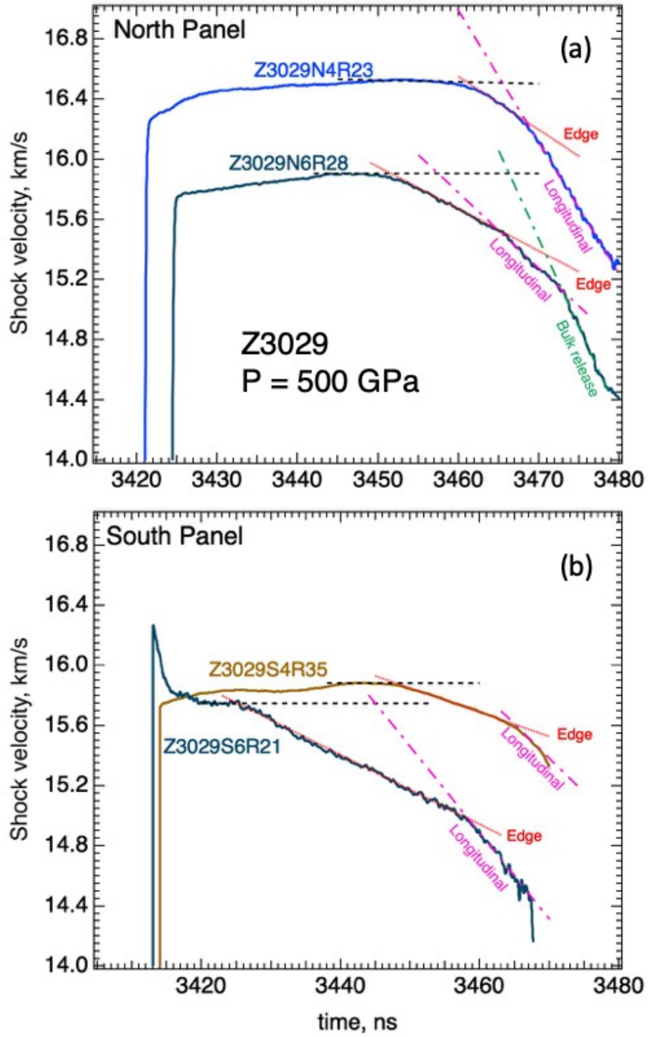

Supplementary Fig. 5. **Wave profiles for shot Z3029 at 500 GPa. (a)** Wave profiles from two samples on the north panel show an edge wave followed by a longitudinal wave. One of the samples also show a bulk release wave that allow us to estimate the bulk sound velocity. **(b)** Wave profiles from two samples on the north panel show the edge and longitudinal waves.

Deleted: S4
Formatted: Font: Bold
Formatted: Font: Bold
Formatted: Font: Bold

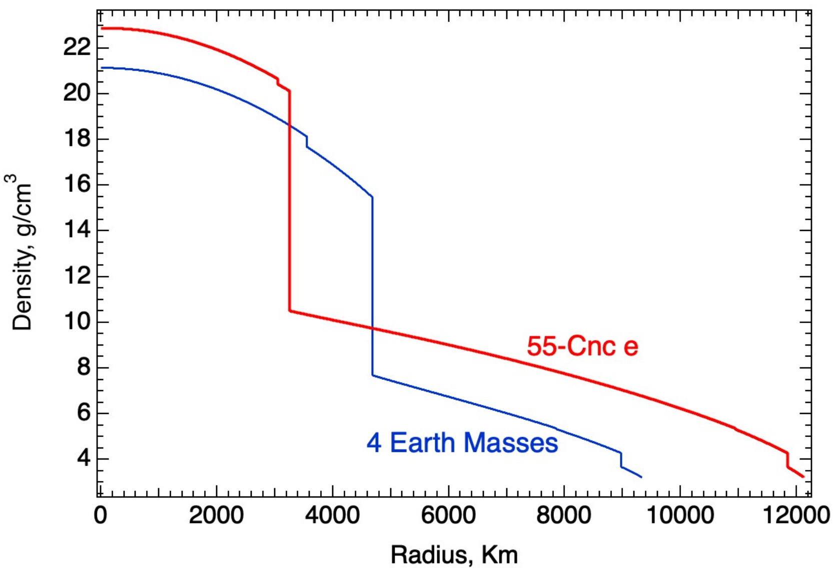

Supplementary Fig. 6. **Calculated density profiles for exoplanet 55 Cnc e (red) and super-Earth with 4 M_E (blue).** The methods for calculating internal structure were described in publication[32]. The first density jump is caused by the transition to bridgmanite. There is a small density jump (~1%) at depth ~1200 Km, caused by the transition to post-perovskite. The post-perovskite mantle density profile is calculated using the parameters optimized in this study. The core density is derived from that of pure iron[30].

Deleted: S5

Formatted: Font: Bold

Deleted: publication[34]

Deleted: iron[28]

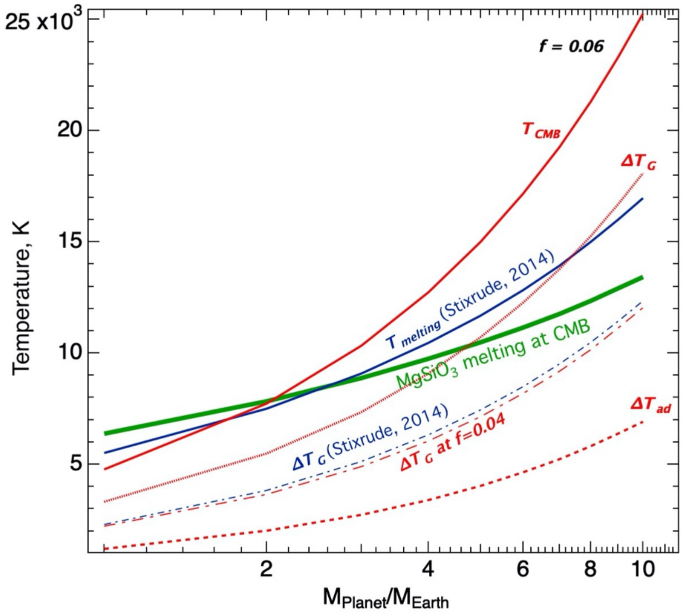

Supplementary Fig. 7. **Calculated initial temperatures at the core-mantle boundary (T_{CMB}) after accretion as a function of mass, with an efficiency factor f = 0.06.** The temperature contribution of the gravitational energy (ΔT_G) and mantle adiabatic compression (ΔT_{ad}) with a mantle potential temperature of 1725 K) are shown. The calculated ΔT_G with an Earth-like f = 0.04 is compared with model of Stixrude[33]. The $MgSiO_3$ melting temperatures of this study are also compared with previous extrapolations[33].

Deleted: S6

Formatted: Font: Bold

Deleted: Stixrude[30]

Deleted: extrapolations[30]

Reviewer #1 (Remarks to the Author):

The authors have carefully responded to my review and made appropriate changes to the manuscript. I do not have any additional concerns. I recommend publication once the other reviewers are satisfied.

Reviewer #2 (Remarks to the Author):

The revised manuscript reasonably addresses all of my suggestions and, in my opinion, the comments provided by the other reviewers. This is a very nice work and I strongly recommend publication of this manuscript.

Reviewer #3 (Remarks to the Author):

The authors convincingly answered to all my comments and questions. I think the revised manuscript now includes all required clarifications, and I am pleased to recommend publication of this very interesting study.
